# Circulating inflammatory proteins associate with response to immune checkpoint inhibition therapy in patients with advanced melanoma

**DOI:** 10.1016/j.ebiom.2022.104235

**Published:** 2022-08-22

**Authors:** Niccolò Rossi, Karla A. Lee, Maria V. Bermudez, Alessia Visconti, Andrew Maltez Thomas, Laura A. Bolte, Johannes R. Björk, Laura Kist de Ruijter, Julia Newton-Bishop, Mark Harland, Heather M. Shaw, Mark Harries, Joseph Sacco, Ruth Board, Paul Lorigan, Elisabeth G.E. de Vries, Nicola Segata, Leonie Taams, Sophie Papa, Tim D. Spector, Paul Nathan, Rinse K. Weersma, Geke A.P. Hospers, Rudolf S.N. Fehrmann, Veronique Bataille, Mario Falchi

**Affiliations:** a Department of Twin Research and Genetic Epidemiology, King's College London, UK; b Centre for Inflammation Biology and Cancer Immunology, King's College London, UK; c Department CIBIO, University of Trento, Trento, Italy; d Department of Gastroenterology and Hepatology, University of Groningen and University Medical Center Groningen, the Netherlands; e Department of Medical Oncology, University of Groningen, University Medical Center Groningen, the Netherlands; f Division of Haematology and Immunology, Institute of Medical Research at St James's, University of Leeds, UK; g Department of Medical Oncology, Mount Vernon Cancer Centre, Northwood, UK; h Department of Medical Oncology, Guy's and St Thomas’ NHS Foundation Trust, London, UK; i Liverpool Clatterbridge Cancer Centre, Liverpool, UK; j Department of Oncology, Lancashire Teaching Hospitals NHS Trust, Preston, UK; k The Christie NHS Foundation Trust, Manchester, UK; l Division of Cancer Sciences, University of Manchester, UK; m School of Cancer and Pharmaceutical Studies, King's College London, UK

**Keywords:** Melanoma, Checkpoint inhibitors, Inflammatory proteins, Response, Survival

## Abstract

**Background:**

Inflammation can modulate tumour growth and progression, and influence clinical response to treatment. We investigated the potential of circulating inflammatory proteins for response stratification of immune checkpoint inhibitor (ICI) therapy for advanced melanoma.

**Methods:**

Study subjects were 87 patients with unresectable stage III or IV cutaneous melanoma from the multiple centres across the United Kingdom (UK) and the Netherlands (NL) who received ipilimumab, nivolumab, or pembrolizumab, or a combination of ipilimumab and nivolumab. Serum samples were collected before and during ICI therapy at follow-up visits scheduled every third week over a 12-week period. We performed targeted quantification of 92 proteins involved in inflammation and tested for association of their pre-treatment and on-treatment levels, as well as longitudinal changes, with overall response rate, progression-free survival, and overall survival.

**Findings:**

We observed consistently higher pre-treatment levels of interleukin-6 (IL-6), hepatocyte growth factor (HGF), and monocyte chemotactic protein 2 (MCP-2), in non-responders compared to responders (meta-analysis *p*=3.31 × 10^−4^, 2.29 × 10^−4^, and 1.02 × 10^−3^, respectively). Patients' stratification according to the median value of IL-6, HGF, and MCP-2 highlighted a cumulative negative effect of pre-treatment levels of the three proteins on response (*p*=1.13 × 10^−2^), with overall response rate among patients presenting with combined elevated IL-6, HGF, and MCP-2 levels being three-fold lower (26.7%) compared to patients with none of the three proteins elevated (80.0%, *p*=9.22 × 10^−3^). Longitudinal data analysis showed that on-treatment changes in circulating inflammatory proteins are not correlated with response.

**Interpretation:**

Our findings are in line with an increasing body of evidence that the pro-inflammatory cytokine IL-6 can influence response to ICI in advanced melanoma, and further support a role of circulating HGF and MCP-2 levels as prognostic biomarkers as suggested by previous smaller studies. Inflammatory proteins may serve as predictive biomarkers of ICI response and valuable targets for combination therapy.

**Funding:**

This work was supported by the Seerave Foundation and Dutch Cancer Society.

**Research in context**

**Evidence before this study**

Immune checkpoint inhibitor (ICI) therapy has revolutionised cancer care, with significant survival advantages observed in multiple tumour types, including melanoma. However, more than 50% of patients still do not achieve sustained clinical long-term benefit from this treatment. Inflammation is a hallmark of cancer and may affect ICI efficacy by steering the anti-tumour response. Growing evidence suggests that inflammatory proteins, and particularly interleukins, can affect tumour response to ICI and could thus be targeted in combination therapy with ICI to maximize the therapeutic effect. However, inflammatory proteins’ contribution to response to ICI in advanced melanoma remains unclear. Additionally, there is a stark paucity of extensive studies of inflammatory proteins in patients undergoing immunotherapy for advanced melanoma.

**Added value of this study**

To our knowledge, this is the most extensive longitudinal study of circulating inflammatory in patients with advanced melanoma treated with single or combination ICI. Our data show that elevated pre-treatment serum levels of IL-6, HGF and MCP-2 are associated with substantially (up to three-fold) lower ICI response rates.

**Implications of all the available evidence**

This study highlights the potential utility of circulating inflammatory proteins to identify patients with advanced melanoma who may benefit most from ICI combined with cytokines antagonists. While large prospective studies are needed to confirm and refine our findings, the results presented here could serve to support clinical decision-making to optimise treatment response.

Alt-text: Unlabelled box

## Introduction

Therapeutic targeting of immune checkpoints such as programmed death-ligand 1 (PD-L1), programmed cell death protein 1 (PD-1), and cytotoxic T-lymphocyte-associated protein 4 (CTLA-4) with immune checkpoint inhibitors (ICIs) has revolutionised the treatment of advanced cutaneous melanoma as well as other tumour types over the past decade. Several landmark randomised trials have shown durable survival benefits, particularly for patients who received combination ICI,^1^ resulting in changes to the standard of care.^2^ Despite these advances, clinical outcomes remain highly variable, and over 50% of treated patients show no beneficial clinical response (*i.e.,* non-responders) or develop resistance to treatment.^3^ Predictive biomarkers for response to ICI are needed to advance future personalised immunotherapies for melanoma. Candidate biomarkers were first reported for tumour cells and in the surrounding immune-micro-environment, and include PD-L1 expression,^4^tumour mutational burden,^5^^,^^6^ presence of lymphoid infiltrates,^7^, ^8^, ^9^and expression levels of interferon (IFN)-γ-inducible genes,^10^among others. However, the clinical applicability of these biomarkers is still debated due to the inconsistency of findings and current limitations associated with the use of tumour biopsies. For this reason, significant effort has been dedicated to the quest for blood-based biomarkers, as peripheral blood sampling is minimally invasive and repeatable, and thus offers a unique opportunity to explore the systemic anti-cancer immunity of the patient throughout the treatment course. At present, proposed host peripheral biomarkers of response to ICI in melanoma include pre-treatment and early on-treatment changes in circulating immune cells composition,^11^, ^12^, ^13^ pre-treatment serum levels of lactate dehydrogenase (LDH),^14^soluble forms of CTLA-4,^15^ PD-1, and PD-L1,^16^ as well as inflammatory proteins^17^^,^^18^ (*i.e*., C-reactive protein (CRP) and interleukins).

In this context, circulating inflammatory proteins, and particularly cytokines, hold the potential to serve as both predictive biomarkers of response to ICI and candidate targets for combination therapy in advanced melanoma. These proteins are involved in tumour development and progression, and regulate host immune activity, promoting the recruitment of immune cells into the tumour microenvironment and regulating the expression of immune checkpoint receptors, including PD-1.^19^

Here, we investigated the relationship between pre-treatment and on-treatment circulating inflammatory proteins and clinical endpoints of response to therapy in a unique dataset of 87 patients with advanced melanoma treated with single or combination ICI. Our data show that pre-treatment levels of interleukin-6 (IL-6), hepatocyte growth factor (HGF) and monocyte chemotactic protein-2 (MCP-2) are additive biomarkers of poor response, and suggest limited capability of on-treatment changes in circulating inflammatory proteins to capture mechanisms of response.

## Methods

### Study subjects

Study subjects were 48 and 39 patients of European ancestry with advanced melanoma from the UK and the Netherlands (NL), respectively, treated with immunotherapy as part of the PRIMM-UK, LEEDS, COLIPI, POINTING, and OncoLifeS studies (Supplementary Methods).

Patient inclusion criteria were: (i) histologically or cytologically confirmed unresectable stage III or stage IV cutaneous melanoma (ii) received treatment with ICI (nivolumab, pembrolizumab, ipilimumab, or a combination of ipilimumab and nivolumab) at a recommended dose as first-line ICI, and (iii) 18 years of age or older. All patients meeting inclusion criteria were offered the opportunity to participate unless it was deemed inappropriate due to advanced age and incapacity, or language difficulties. Patients were followed up in their respective cancer centres as per standard-of-care, with research nurses ensuring appropriate capture of survival data.

### Ethics

The PRIMM-UK study was approved by the South Central Berkshire committee of the Research Ethics Services (RES) of the NHS. The LEEDS study was approved by the North West - Greater Manchester West Research Ethics Committee with reference number 15/NW/0933. The COLIPI, POINTING, and OncoLifeS studies have been approved by the Medical Ethical Committee (in Dutch: Medisch Ethische Toetsingsingscommissie or METc) of the University Medical Center Groningen (UMCG) in the Netherlands. Written informed consent was obtained from all patients.

### Clinical outcomes

Response to ICI was defined based on radiological evaluation according to Response Evaluation Criteria in Solid Tumours (RECIST) v1.1 criteria (Supplementary Methods).^20^ Patients were classified as responders (including complete response, partial response, or stable disease at radiological evaluation approximately six months after ICI initiation) or non-responders (progressive disease). Clinical endpoints included overall response rate (ORR), progression-free survival (PFS, *i.e.*, the time from the first dose of ICI to progressive disease or death from any cause), and overall survival (OS, *i.e.*, the time from the first dose of ICI to death). To include late responders in our analysis, patients with progressive disease on the first radiological evaluation but a response at the second radiological evaluation compared to baseline imaging were also labelled responders. Patients with progressive disease on the first radiological evaluation that was confirmed on the next follow-up scan, or patients with progressive disease on the first radiological evaluation that were unable to complete a confirmation scan due to clinical progression or death were labelled non-responders.

### Sample collection

Serum samples were collected before (*i.e.*, baseline samples) and during ICI (*i.e.*, follow-up samples) approximately every third week after ICI initiation (Supplementary Figure 1). Follow-up samples were grouped according to the time from the start of treatment into i) early follow-up samples, comprising samples collected within 2–5 weeks after ICI initiation (typically before 2^nd^ ICI cycle) and ii) late follow-up samples, comprising samples collected within 5–12 weeks after ICI initiation (typically after 2^nd^ ICI cycle; Supplementary Figure 1). When multiple samples were available within the same time window, the sample collected at the follow-up visit closest to the window midpoint (*i.e.*, 3 and 9 weeks for early and late follow-up windows, respectively) was used.

### Inflammatory proteins measurement

We used the Proximity Extension Assay technology (Olink Inflammation panel, Olink® Proteomics) to measure 92 inflammatory proteins in serum.^21^Data was pre-processed by Olink using Olink® NPX Manager software, and provided in an arbitrary log_2_-scaled unit (Normalised Protein eXpression, NPX) defined by normalising and log_2_-transforming the Ct (cycle threshold) values obtained from the quantitative polymerase chain reaction (qPCR). Proteins with >25% of the samples below Olink predetermined lower detection limit (LOD) were discarded (n=15; Supplementary Figure 2). For remaining proteins, values below the LOD were removed. In addition, we excluded extreme values exceeding a relaxed threshold of four standard deviations (SD) from the protein sample mean to reduce the risk of bias in the association test statistic while avoiding overfiltering. According to Chebyshev's theorem, up to ∼5% of the data may exceed four SD from the sample mean from any arbitrary distribution, thus leaving at least ∼95% of the data for subsequent analysis. Additional details about the 92 markers and data pre-processing are available at https://www.olink.com/resources-support/document-download-center/. To assess the presence of sample stratification, we performed principal component analysis (PCA) on proteomic data.

### Statistics

Data processing and analysis were done in R (v4.1.2).

Primary patients’ characteristics were summarised using frequencies and proportions for categorical variables, and using median, interquartile range (IQR), and range for continuous variables. We used Fisher's exact test and Kruskal-Wallis’ test to compare categorical and continuous variables, respectively, between study centres and between responders and non-responders. *P*-values <0.05 were considered significant.

### Association between pre-treatment inflammatory proteins and response rate

To identify predictive biomarkers of response to ICI, mean baseline differences of the 77 inflammatory proteins (mean log_2_-FC) between responders and non-responders were estimated in patients from the UK and the NL, along with their respective standard deviations, and supplied to a random-effects meta-analysis model using the restricted maximum-likelihood estimator (*rma* function, metafor R package, v3.4-0). Due to the correlation between inflammatory proteins (Supplementary Figure 3), we used Li's method^22^ to estimate the effective number of independent tests, in order to correct for multiple hypothesis testing. The derived random-effects *p*-value threshold for statistical significance was 0.05/42 = 1.19 × 10^−3^.

### Response rate prediction and ROC

We used the least absolute shrinkage and selection operator (LASSO) to estimate the ability of pre-treatment levels IL-6, HGF, and MCP-2 to discriminate between responders and non-responders. Two nested models were trained in the UK sample, namely (i) a full model, where pre-treatment IL-6, HGF, and MCP-2 levels, as well as clinical/demographic variables associated with ORR (namely sex, age, BMI, and metastatic stage), were included as predictors, and (ii) a null model, including only the clinical/demographic variables. For each model, the λ parameter was selected to minimise the cross-validation prediction error using a leave-one-out cross-validation (LOOCV) setting (glmnet R package v4.1-4). Model performance was evaluated by means of the AUC-ROC (area under the receiver operating characteristic curve) obtained in the NL sample. We used permutation testing to evaluate the contribution of IL-6, HGF, and MCP-2 to the full model performance over the null model. More in detail, we generated 1000 random permutations of the test set (*i.e.*, NL sample) where labels indicating protein levels were randomly permuted as a single set, thus preserving their natural correlation structure. Conversely, age, sex, BMI, and metastatic stage, were not permuted. Next, we estimated the AUC-ROC of the original full model for each random reshuffling of the test set, thus providing a null distribution for the performance of the model based on pre-treatment IL-6, HGF, and MCP-2 levels to predict ORR. An empirical p-value was estimated as the probability of observing an AUC-ROC larger than the one observed in the original test set. An empirical *p*-value <0.05 was considered significant.

### Longitudinal analyses of pre- and on-treatment inflammatory proteins

We exploited the longitudinal measurements of circulating inflammatory proteins to investigate: (i) the overall effect of ICI on inflammatory protein levels independently from response to treatment (*i.e.*, paired pre-post ICI initiation in combined responders and non-responders), (ii) the association between longitudinal changes of inflammatory protein levels (*i.e.*, change scores) and ORR, and (iii) the behaviour throughout the treatment course of the pre-treatment inflammatory proteins showing association with ORR at the baseline. Statistical differences were determined using two-sided Wilcoxon tests. For each follow-up time window (*i.e.*, 2–5 and 5–12 weeks post-ICI initiation), protein change scores were obtained by subtracting measurements taken at follow-up from those taken at baseline. We implemented a multivariate permutation test (MPT) to compute statistical significance while controlling for family-wise error rate resulting from multiple comparisons (Supplementary Methods).^23^ For each follow-up time window, empirical *p*-values were obtained using MPT based on 5,000 permutations of the dataset. Empirical *p*-values <0.05 were considered significant. Correlation of longitudinal changes of serum proteins following ICI initiation between responders and non-responders was assessed by means of the Pearson correlation between the change scores of the 77 inflammatory proteins at 2–5 (early follow-up) and 5–12 weeks (late follow-up) post-ICI initiation.

### Association between inflammatory proteins, and PFS and OS

We examined the correlation between pre-treatment inflammatory proteins associated with ORR at baseline and PFS and OS using the log-rank test (*logrank.test* function, nph R package, v2.0) on dichotomised protein levels (*i.e.*, low vs high) at the median expression value. Survival curves were estimated using the Kaplan–Meier method (*survfit* function, survival R package, v3.2-13). To assess the significance of the log-rank statistics empirical p-values were estimated using MPT based on 5000 permutations, separately for OS and PFS. Empirical *p*-values <0.05 were considered significant.

### Quantitative estimation of serum biomarkers using Luminex

To provide clinically-interpretable estimates of the magnitude of the effect of inflammatory proteins on clinical endpoints of response to ICI, we quantified the absolute pre-treatment serum concentration of inflammatory proteins associated with ORR at baseline using a custom Luminex-based assay (Luminex, Austin, TX, USA; Supplementary Methods). Spearman correlation was used as a measure of concordance between Olink and Luminex data.

Association between Luminex-derived protein levels and ORR was assessed using logistic regression (*glm* function, stats R package, v4.1.1). Sex, age, body mass index (BMI)*,* and metastatic stage were included as covariates in the analysis. Confidence intervals for regression coefficients were obtained by fitting the same model on 1000 simulated datasets of equal size obtained by randomly sampling the original dataset with replacement. *P*-values <0.05 were considered significant.

Survival analysis was performed using multivariate Cox proportional hazards regression on Luminex data (*coxph* function, survival R package, v3.2-13). Age, sex, BMI, metastatic stage, and LDH levels were included as covariates. To assess violation of the proportional hazards assumption, we used the *cox.zph* function (survival R package, v3.2-13) to obtain the global χ^2^ statistic for each model. We considered the assumption as met if the global χ^2^ *p*-value was >0.05.

### Role of funders

The funders had no role in the study design, data collection, data analysis, data interpretation, or writing of this report.

## Results

Study subjects were 87 previously ICI-naive patients with unresectable stage III or IV melanoma undergoing ICI treatment from the UK (*n*=48) and the NL (*n*=39; Table 1; Supplementary Figure 1). The average age was 62 years, and 66% of the patients (*n*=57) were males (Table 1). Forty-six patients were classified as responders and 41 as non-responders, corresponding to an ORR of 52.9%. Patients’ sex, age, BMI, LDH levels, ECOG-PS, BRAF mutational status, and ORR were similar across the UK and the NL sample (*p*>0.05). While patients’ age did not associate with ORR, patients’ sex was associated with ORR and OS, with males having lower odds of positive response to ICI compared to females (odds ratio, (OR)[95% CI]=0.27[0.09–0.76]; Fisher *p*=6.90 × 10^−3^), as well as shorter survival (HR[95% CI]=2.18[1.11–4.30]; log-rank *p*=0.046). Additionally, BMI showed a weak negative association with response to ICI, after accounting for age and sex (OR[95% CI]=0.91[0.82–0.99], *p*=4.66 × 10^−2^).

**Table 1:** Demographic characteristics, treatment details, and response by RECIST v1.1 of 87 patients with advanced melanoma from the UK and the NL.

	All	UK	NL	*p*	*p*_ORR_	*p*_OS_
Clinical details						
N	87	48	39			
Sex (males)	57 (65.5%)	35 (72.9%)	22 (56.4%)	0.119	<0.01	<0.05
Age	62 [53–73]	63 [52–80]	59 [54–67]	0.235	0.459	0.134
BMI (kg/m^2^)	27.1 [24.4– 32.0]	28.2 [24.5– 32.1]	26.4 [24.1–30.6]	0.234	<0.05	0.389
Tumour BRAF mutant	40 (46.5%)	17 (36.2%)	23 (59.0%)	0.050	0.387	0.844
Elevated LDH	30 (34.5%)	20 (41.7%)	10 (25.6%)	0.173	0.259	<0.01
Metastatic stage						
III unresectable	5 (5.7%)	4 (8.3%)	1 (2.6%)	<0.001	<0.05	<0.05
IV M1a	12 (13.8%)	10 (20.8%)	2 (5.1%)			
IV M1b	15 (17.2%)	12 (25.0%)	3 (7.7%)			
IV M1c	32 (36.8%)	18 (37.5%)	14 (35.9%)			
IV M1d	23 (26.4%)	4 (8.3%)	19 (48.7%)			
ECOG performance status						
0	46 (52.9%)	21 (43.8%)	25 (64.1%)	0.190	0.287	0.114
1	32 (36·8%)	22 (45.8%)	10 (25.6%)			
2	7 (8.0%)	4 (8.3%)	3 (7.7%)			
3	2 (2.3%)	1 (2.1%)	1 (2.6%)			
Treatment						
Nivolumab & ipilimumab	39 (44.8%)	27 (56.2%)	12 (30.8%)	<0.01	0.831	0.089
Pembrolizumab	21 (24.1%)	14 (14.6%)	7 (17.9%)			
Nivolumab	26 (29.9%)	7 (29.2%)	19 (48.7%)			
Ipilimumab	1 (1.1%)	0 (0.0%)	1 (2.6%)			
Outcome						
Responders	46 (52.9%)	23 (47.9%)	23 (59.0%)	0.389	*n.a*.	<0.001
Responders classification						
Complete response	6 (13.0%)	5 (21.7%)	1 (4.3%)	0.068	*n.a.*	*n.a.*
Partial response	25 (54.3%)	14 (60.9%)	11 (47.8%)			
Late response	3 (6.5%)	0 (0.0%)	3 (13.0%)			
Stable disease	12 (26.1%)	4 (17.4%)	8 (34.8%)			

For continuous variables, median and IQR range (within brackets) are shown, whereas absolute count and percentage (within parentheses) are shown for categorical variables. P-values of the association between clinical variables and study centre (*p*), ORR (*p*_ORR_), and OS (*p*_OS_) are shown. Differences between study centres were tested using Kruskal-Wallis’ test (for continuous variables) and Fisher's exact test (for categorical variables). Association between age and ORR and OS was evaluated using Kruskal-Wallis’ test and log-rank test, respectively. For the log-rank test, age was dichotomized at the median value. Associations between sex, LDH levels, BRAF mutation status, ECOG-PS (ECOG-PS=0 vs ECOG-PS≥1), and treatment type (single vs combined) were assessed using Fisher's exact test and log-rank test for ORR and OS, respectively. Association between BMI and ORR and OS was assessed using logistic regression and Cox regression, respectively, while accounting for patients’ age and sex. Association between metastatic stage and ORR and OS was assessed using logistic regression and Cox regression, respectively, while accounting for patients’ age, sex, and BMI.

We observed differences across the UK and NL sample with respect to: (i) the proportion of patients receiving single or combination therapy (Fisher *p*=1.66 × 10^−3^), with patients from UK receiving mainly a combination of ipilimumab and nivolumab (56.2%), while a large fraction (48.7%) of patients from NL receiving nivolumab alone, and (ii) tumour stage, with patients from UK showing overall a lower prevalence of M1d disease (8.3%) compared to patients from NL (48.7%, Fisher *p*=9.70 × 10^−5^).

In the combined sample, BRAF mutation status and ECOG-PS (ECOG-PS=0 vs ECOG-PS≥1) were not associated with either ORR or OS (*p*>0.05), while higher cancer stage was associated with both poorer ORR (OR[95% CI]=0.62[0.39–0.94]; *p*=0.030) and OS (HR[95% CI]=1.52 [1.05–2.19]; *p*=0.026), after accounting for age, sex and BMI. Elevated LDH levels were negatively associated with OS (HR[95% CI] = 2.38[1.14–4.97]; log-rank *p*=7.82 × 10^−3^), but not with ORR (*p*>0.05). Combination therapy did not associate with improved ORR or OS over monotherapy, likely due to the small sample size.

Baseline serum samples were collected from the 87 patients on average 7 days before starting ICI (median[IQR]=2[0-6] days before ICI initiation). Follow-up serum samples were available for 56 patients. These included 46 patients (19 non-responders and 27 responders) with a follow-up sample taken 2–5 weeks after starting the treatment (*i.e.*, early follow-up window, mainly comprising samples collected before starting the 2^nd^ ICI cycle) and 35 patients (11 non-responders and 24 responders) with a follow-up sample available within 5–12 weeks from ICI start (*i.e.,* late follow-up window, mainly comprising samples collected after starting the 2^nd^ ICI cycle). Serum samples were used to measure the expression levels of 92 inflammatory proteins (Olink Inflammation panel). Of the 92 proteins, 15 were below the assay limit of detection in >25% of the samples and were therefore discarded, leaving 77 proteins for subsequent analyses (Supplementary Figure 2). We did not find evident sample stratification based on proteomic data due to study centres or plate effects (Supplementary Figure 4).

**Figure 1:**
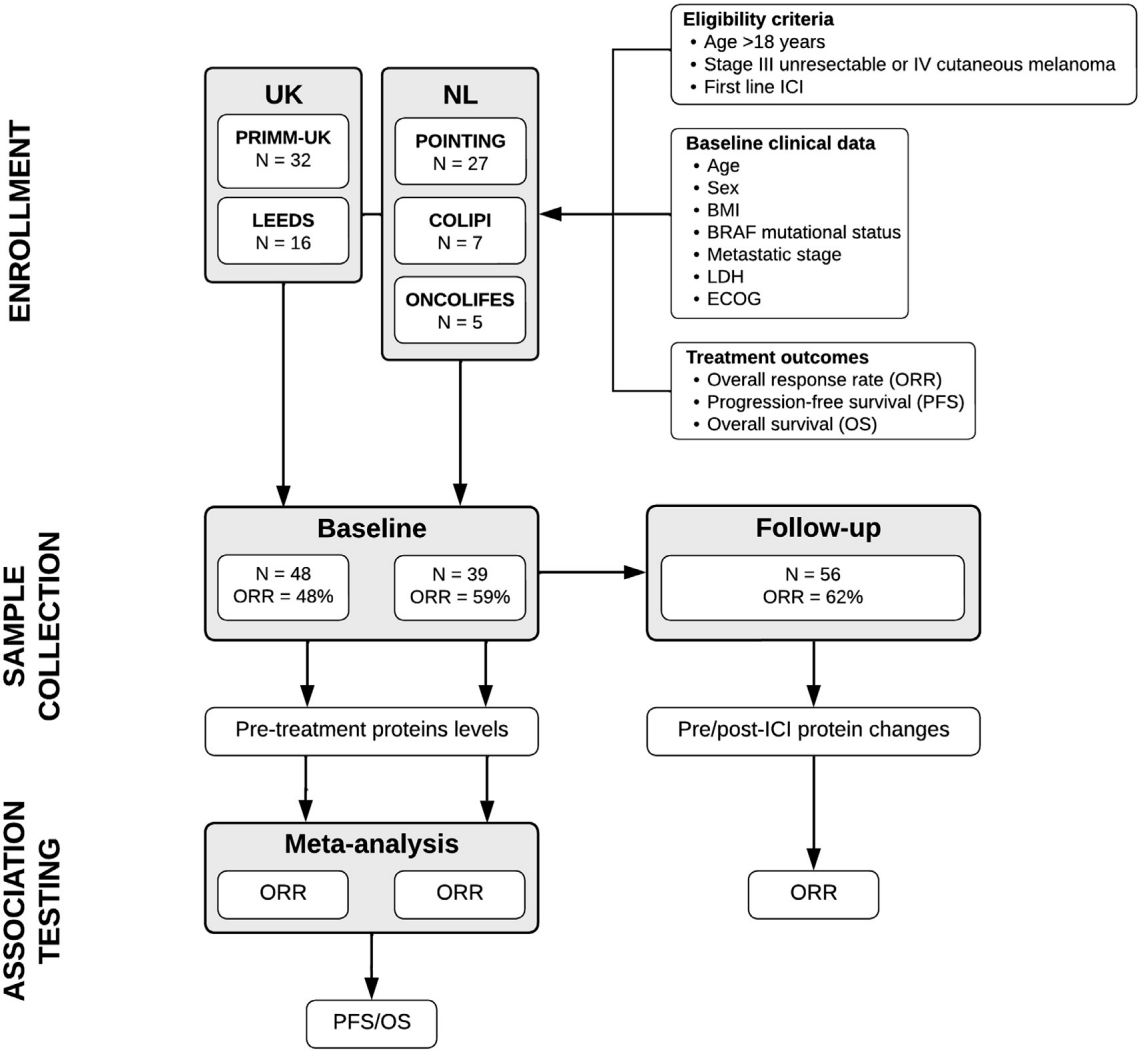
Flow diagram of the progress through phases of the analysis.

**Figure 2:**
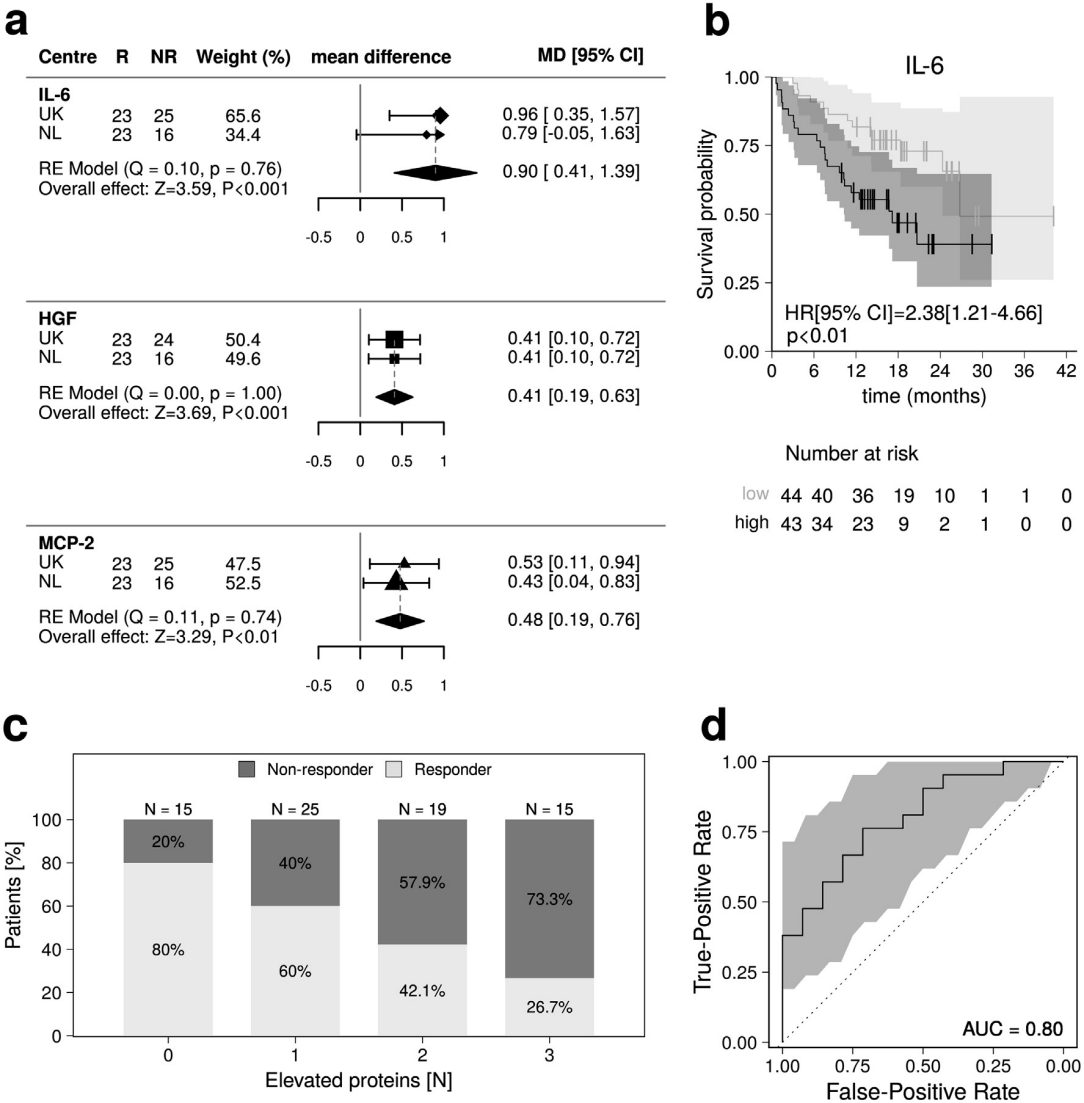
**(a)** Forest plots of the meta-analysis showing the mean difference between responders and non-responders of the three pre-treatment inflammatory proteins (Olink data) that associate with ORR. For each of the two datasets (*i.e.*, UK and NL), we report the number of responders (R) and non-responders (NR), the dataset weight in the random-effects meta-analysis, the mean protein difference (log_2_-FC), along with its 95% CIs. Pooled mean effect sizes, with 95% CIs and *p*-values are also shown, as well as the statistics of Cochran tests for heterogeneity. **(b)** Survival curves in the 87 patients with advanced melanoma presenting with high (black line) and low (grey line) pre-treatment IL-6 levels, as measured using the Olink assay. Vertical lines indicate censored data points. Log-rank test hazard ratio (HR), along with the 95% CI and *p*-value, is shown. **(c)** Bar plot showing response rates in the 87 patients with advanced melanoma, stratified according to the number of inflammatory markers of response (*i.e*., IL-6, HGF, and MCP-2, as measured using the Luminex assay) being elevated (*i.e.*, above the median) at baseline. **(d)** AUC-ROC curve showing ORR prediction accuracy, as estimated by training a LASSO logistic regression model using LOOCV in the UK sample and using the NL sample as validation set. Variables included in the LASSO model were age, sex, BMI, metastatic stage, IL-6, HGF, and MCP-2 (Olink data). AUC, area under the curve; LOOCV, leave one out cross-validation.

We first looked for inflammatory proteins consistently associated with response to ICIs across patients from the UK and the NL in relation to ORR (Figure 1). Pre-treatment levels of IL-6, HGF, and MCP-2 were higher in non-responders compared to responders in the meta-analysis (Figure 2-a), with the pooled estimated mean log_2_-FCs [95% CIs] being 0.90[0.41–1.39] (*p*=3.31 × 10^−4^), 0.41[0.19–0.63] (*p*=2.29 × 10^−4^), and 0.48[0.19–0.76] (*p*=1.02 × 10^−3^), respectively (Figure 2-a; Supplementary Table 1). We didn't observe evidence of heterogeneity between UK and NL in terms of IL-6, HGF, and MCP-2 effects on ORR (Cochran's Q *p*>0.05; Figure 2-a; Supplementary Table 1). Elevated pre-treatment IL-6 levels were also associated with shorter OS (log-rank *p*=9.95 × 10^−3^; Figure 2-b).

To explore underlying effects of different ICI regimens on proteins’ association with ORR, we assessed the association between pre-treatment serum levels of IL-6, HGF, or MCP-2 and ICI agent (single vs combined) but found no evidence of association at an alpha threshold of 0.05.

LASSO regression was performed to estimate the prediction ability of IL-6, HGF, MCP-2 to segregate responders and non-responders (Methods). We achieved an AUC-ROC of 0.80 in test set when IL-6, HGF, and MCP-2 were included as predictors, together with age, sex, BMI, and metastatic stage (Figure 2-d). The AUC-ROC in the test set dropped to 0.69 when the three cytokines were excluded from the set of predictors. Permutation analysis confirmed that IL-6, HGF, MCP-2 have a relevant contribution over clinical/demographic variables to discriminate between responders and non-responders (*p*_emp_=4.00 × 10^−3^).

Next, we explored the contribution of ICI on dynamic inflammatory protein changes irrespective of response status. In combined responders and non-responders, we observed a significant increase from baseline in follow-up levels of TNF receptor superfamily member 9 (TNFRSF9; mean FC=1.43, paired Wilcoxon *p*=1.57 × 10^−10^) and CXC motif chemokine ligand 9 (CXCL9; mean FC=2.41, *p*=5.81 × 10^−8^) at the early follow-up window (Supplementary Table 3; Supplementary Figure 6). CXCL9 levels also increased from baseline at the late follow-up window (mean FC=2.51, *p*=5.26 × 10^−8^; Supplementary Table 3, Supplementary Figure 6). Subsequently, we investigated if dynamic changes in circulating inflammatory proteins following ICI initiation reflect immunological mechanisms of response to ICI, and thus tested the association between longitudinal variations of the 77 inflammatory proteins and ORR. There was no difference between responders and non-responders in terms of protein change from baseline at either early or late follow-up windows (*p*_emp_>0.05, Supplementary Table 4), with protein change scores being correlated between responders and non-responders at both time windows (Pearson_early_ *ρ*=0.71, *p*=6.54 × 10^−13^; Pearson_late_ *ρ*=0.64, *p*=4.88 × 10^−10^; Supplementary Figure 7). In line with these observations, inflammatory proteins associated with ORR at baseline remained consistently higher in non-responders compared to responders also at follow-up (Supplementary Figure 8). IL-6, HGF, and MCP-2 levels were higher in non-responders compared to responders at the early follow-up window (mean FC = 1.24, 1.27, and 1.35, Wilcoxon *p* = 2.32 × 10^−2^, 2.75 × 10^−3^, and 3.31 × 10^−2^, respectively; Supplementary Table 5), and IL-6 levels were elevated at the late follow-up window (mean FC=1.60, *p*=1.33 × 10^−2^; Supplementary Table 5*;* Supplementary Figure 8).

To validate our findings and improve their clinical interpretability, we used a custom Luminex-based assay to quantify the absolute pre-treatment serum concentration of IL-6, HGF, and MCP-2, and calculate estimates of their effect on ORR per picograms/millilitre (pg/mL) increase. Additional serum samples were available at baseline for 81 out of 87 patients, including 41 responders and 40 non-responders, corresponding to 93% of the subjects included in the Olink assay analysis. Protein measures were highly concordant between the Olink and Luminex assays (Spearman's *ρ*=0.69–0.89, *p*<2.10 × 10^−12^; Supplementary Figure 5). Serum level ranges of IL-6, HGF, and MCP-2 were 0.20–19.21, 32.44–536.72, and 14.83–117.89 pg/mL, respectively, and the estimated ORs for ORR per pg/mL increase in serum protein level were 0.76 (95% CI=0.32–0.90, *p*=7.70 × 10^−3^), 0.99 (95% CI=0.97–0.99, *p*=2.78 × 10^−3^), and 0.97 (95% CI=0.93–1.00, *p*=3.85 × 10^−2^), respectively (Supplementary Table 2), after accounting for age, sex, BMI, and metastatic stage. As pre-treatment IL-6 also showed an association with OS, we performed multivariate Cox regression analysis on the Luminex data to estimate the contribution of circulating IL-6 levels on patients' survival. The resulting hazard ratio for the rate of death per pg/mL increase in IL-6 was 1.90 (95% CI=1.32–2.73, *p*=5.46 × 10^−4^) after accounting for age, sex, BMI, metastatic stage, and LDH levels.

Of note, while we observed higher pre-treatment levels of IL-6 among patients with elevated LDH levels or ECOG-PS≥1 compared to patients with normal LDH levels or ECOG-PS=0 (Wilcoxon *p*=2.11 × 10^−4^ and 1.44 × 10^−4^, respectively; Supplementary Figure 9), IL-6 showed consistent association with ORR when LDH and ECOG-PS were added as covariates to multivariate logistic regression model (*p*<0.05). Similarly higher pre-treatment IL-6 levels were associated with shorter survival when ECOG-PS was added as covariates to the multivariate Cox regression models (*p*<0.01).

Lastly, to further evaluate the capability of pre-treatment IL-6, HGF, and MCP-2 levels to discriminate between responders and non-responders, we stratified patients according to the median value of the distributions of these proteins (Luminex data) in our sample. Median[IQR] pre-treatment levels of IL-6, HGF, and MCP-2 in the lower half were 0.90[0.67–1.19], 147.17[120.21–169.92], and 40.85[33.45–48.15] pg/mL, respectively, while in the upper half were 2.29[1.82–4.92], 247.22[218.89–300.47], and 68.11[60.14–85.68] pg/mL, respectively. Prevalence of elevated (*i.e.*, above the median) pre-treatment levels of IL-6, HGF, and MCP-2 among responders was 36.6%, 36.6%, and 35.9%, respectively, while among non-responders was 64.1% (Fisher *p*=2.47 × 10^−2^), 64.9% (*p*=2.27 × 10^−2^), and 64.1% (*p*=2.29 × 10^−2^), respectively (Supplementary Figure 10). ORR exhibited a linear decrease with increasing number of these inflammatory proteins being elevated: ORR in patients in the upper half of all the three proteins (26.7%) was three-fold lower compared to patients with below-median levels of all three proteins (80.0%, Fisher *p*=9.22 × 10^−3^, Figure 2-c), while patients with above-median levels of two or only one protein(s) showed intermediate response rates of 42.1% and 60.0%, respectively (Figure 2-c). Notably, we found evidence for an additive effect of elevated pre-treatment levels of IL-6, HGF, and MCP-2 on decreased ORR (OR[95% CI]=0.45[0.23–0.81]; *p*=1.13 × 10^−2^), as estimated by regressing ORR on the total number of elevated proteins, and accounting for age, sex, BMI, and metastatic stage.

## Discussion

We have conducted, to the best of our knowledge, the largest longitudinal study to date exploring the relationship between multiple circulating markers of inflammation and response to therapy in patients with advanced melanoma undergoing both single-agent (55%) and combination (45%) ICI.

Higher pre-treatment levels of either IL-6, HGF, or MCP-2 were consistently associated with lower ICI response rates, with the estimated OR[CI] per pg/mL increase being 0.76[0.32–0.90], 0·99[0.97–0.99], and 0.97[0.93–1.00], respectively. Pre-treatment IL-6 levels were also negatively associated with patients’ OS, with a resulting hazard ratio[CI] per pg/mL increase of 1.90[1.32–2.73].

IL-6 is a key pleiotropic immunomodulatory cytokine secreted by both normal and tumour cells, and its role in inflammatory-associated carcinogenesis, tumour growth, and angiogenesis has been extensively described.^24^ Elevated pre-treatment IL-6 levels have been associated with reduced ICI response rates by two independent studies on unresectable stage III or IV melanoma, including a study of 35 patients treated with nivolumab,^25^and a larger study of 140 patients treated with sequential administration of nivolumab followed by ipilimumab (or vice versa).^17^

HGF is a multi-functional factor involved in cell growth, cell motility, and morphogenesis, and has been reported to be involved in cancer by promoting angiogenesis, tissue regeneration, tumourigenesis and metastasis.^26^ In transgenic mice overexpressing HGF, ultraviolet-B exposure promoted the early appearance of rapidly enlarging primary melanomas, showing enhanced invasive and metastatic behaviour.^27^ Negative association between serum HGF and ORR has been previously observed in 29 Japanese patients with metastatic melanoma receiving either nivolumab or pembrolizumab,^28^ and the present study confirms the validity of this association in a larger sample of European ancestry.

MCP-2 functions in a wide variety of inflammatory cells as a chemotactic factor,^29^ and previous work has indicated an oncogenic role for MCP-2 by fostering a prometastatic environment.^30^,^31^ A study in 28 patients with metastatic melanoma previously reported that baseline melanoma biopsies from ICI-resistant patients display a higher expression of the MCP-2 coding gene (i.e., *CCL8*) compared to responding baseline tumours.^32^ The present study reports that also circulating MCP-2 levels are higher in non-responders compared to responders, thus further supporting its potential role in response to ICI.

In our sample, patients with combined elevated (*i.e.*, above the median) levels of IL-6, HGF, and MCP-2 at baseline were three times less likely to respond to therapy than patients with combined low (*i.e.*, below the median) levels. Overall, our data provide further support for a role of HGF and MCP-2 as candidate biomarkers of response to ICI in advanced melanoma, while proposing an additive negative effect of these two cytokines, together with IL-6, on response rates.

Interestingly, median IL-6 levels in patients in the upper half of pre-treatment IL-6 distribution (IL-6>1.57, median=2.29 pg/mL) roughly corresponds to the cutoff value for high serum IL-6 (IL-6>2 pg/mL) proposed by a large (*n*=3044) population-based study and associated with higher risk of unhealthy ageing and death from non-cardiovascular causes.^33^ Response rate in the whole sample was 53% which increased to 65% in subjects with low IL-6 levels (*i.e.*, below the median; median=0.90 pg/mL). Similar to IL-6, subjects with low pre-treatment HGF or MCP-2 levels (*i.e.*, below the median; HGF<188.98 pg/mL, median=147.17 pg/mL; MCP-2<50.99 pg/mL, median=40.85 pg/mL) showed response rates of 67% and 64%, respectively. Interestingly, preliminary results presented at the ESMO Congress 2021 from an ongoing Phase II clinical trial in 41 patients with unresectable melanoma treated with a combination of ipilimumab, nivolumab and anti-IL-6 antibodies reported an encouraging 60% response rate.^34^

We provide evidence for an additive negative effect of IL-6, HGF, and MCP-2 on response rate in patients with advanced melanoma receiving either single or combination ICI therapy, with patients with combined elevated (*i.e.*, above the median) levels of the three inflammatory proteins at baseline being three times less likely to respond to therapy than patients with combined low (*i.e.*, below the median) levels.

Furthermore, we showed that on-treatment changes in circulating inflammatory proteins occur within three months following ICI initiation in all patients (responders and non-responders), with TNFRSF9 and CXCL9 undergoing pronounced increases. However, response to ICI was not associated with a distinctive longitudinal profile for any of the 77 inflammatory proteins, and on-treatment IL-6, HGF, and MCP-2 levels were consistently higher in non-responders compared to responders.

In contrast with our findings, association between on-treatment variation of serum CXCL9 and response to ICI has been observed in a study of 28 patients with advanced melanoma.^35^ While this discrepancy may be imputable to heterogeneity between studies, on-treatment changes involving soluble immune mediators (including CXCL9 transcriptional changes) associated with response to ICI in patients with advanced melanoma have been mainly reported within the tumour microenvironment by previous studies.^36^ While future work leveraging larger sample sizes or more comprehensive inflammatory biomarkers panels may elucidate mechanisms of response to ICI involving dynamic changes in circulating inflammatory proteins, poor correlation between local (*i.e.*, within the tumour microenvironment) and peripheral levels of soluble immune mediators may explain the lack of association between on-treatment changes in circulating inflammatory proteins and response to ICI reported by the present study. In addition, as response to ICI in patients with advanced melanoma has been associated with on-treatment changes in peripheral immune cell composition by multiple studies,^12^^,^^13^ circulating cytokine levels may not accurately reflect the antitumour activity of the specific immune cell subpopulations mediating response to ICI, as the same cytokine can be secreted by different immune cell types.

The present study has some limitations. First, the sample size and the lack of an external validation sample which both limit the conclusions about the clinical applicability of our findings. Randomised prospective studies assessing differences in clinical outcome in patients with advanced melanoma according to high/low cytokines levels (*i.e.*, biomarker-stratified study design) are needed to confirm the role of IL-6, HGF, and MCP-2 as potential predictive biomarkers of ICI efficacy for clinical use. Second, study participants were mainly aged individuals (median age at primary melanoma diagnosis = 57), and predominantly males (66%, proportion test *p*<0.01). While this dataset may be considered representative of the general melanoma patient population based on previous reports on average age at melanoma diagnosis,^37^, ^38^, ^39^ as well as the higher incidence rate of melanoma observed in aged males compared to females,^40^ larger studies with more balanced sex ratio, as well as younger patients, are needed to examine sex- and age-differences on IL-6, HGF, and MCP-2 contribution to clinical response to ICI. Third, while we observed comparable pre-treatment IL-6, HGF, and MCP-2 levels between patients treated with either single or combined ICI, further studies are needed to explore possible interactions between different ICI regimens and inflammatory proteins on response. While our data suggest that IL-6, HGF, and MCP-2 retain independent association with ORR, the nature of the relationship between these three inflammatory proteins in the context of advanced melanoma remains to be elucidated. For example, previous work on macrophages isolated from mice showed that IL-6 and HGF may be linked by a negative feedback loop,^41^ suggesting possible causal influences between IL-6 and HGF underlying their mutual association with ORR.

In conclusion, we provide evidence of an additive negative effect of IL-6, HGF, and MCP-2 on clinical response to single or combination ICI in advanced melanoma. These findings may help select patients who would benefit from combination therapy with ICI and cytokines antagonists, and represent a further step towards a better understanding of drug response for future individualized antitumour immunotherapy of advanced melanoma.

## Contributors

NR, VB, and MF designed the study. LAB, LKdR, JNB, MHarland, HMS, MHarries, JS, RB, JS, PL, EGEdV, PN, RKW, and GAPH were involved with sample and clinical data collection and/or curation of the metadata. KAL, LAB and LKdR verified the underlying data. NR processed the raw proteomic data and carried out the computational analyses with contribution from RSNF and AV. MVB ran the Luminex assay under the supervision of SP and LT. NR and KAL interpreted the results. NS, AMT, JRB, TDS and MF supported the interpretation of data, analysis and results. NR and KAL wrote the manuscript. All authors read and approved the final version of the manuscript.

## Data sharing statement

Individual-level participant data used in this study can be provided to researchers upon request. A detailed proposal for how the data will be used is required.

## Declaration of interests

RKW acted as a consultant for Takeda, received unrestricted research grants from Takeda, Johnson & Johnson, Tramedico, and Ferring, and received speaker fees from MSD, Abbvie and Janssen Pharmaceuticals. TDS is a co-founder of Zoe Global. EGEdV reports an advisory role at Daiichi Sankyo, NSABP and Sanofi (paid to University Medical Center Groningen), and research funding from Amgen, AstraZeneca, Bayer, Crescendo, Chugai Pharma, CytomX Therapeutics, G1 Therapeutics, Genentech, Nordic Nanovector, Radius Health, Regeneron, Roche, Servier and Synthon (paid to University Medical Center Groningen). SP received speaker fees from Almirall, BMS, ISDIN, La Roche Posay, Leo Pharma, Regeneron, Roche, Sanofi, and acted as an advisory board for Almirall, ISDIN, La Roche Posay, Pfizer, Roche, Regeneron, Sanofi, Sunpharma and research funding from Abbie, AMGEN, ISDIN, La Roche Posay, Leo Pharma, Novartis. SP is also an employee of Enara Bio. RB has received honoraria from, and sits on advisory boards of, Novartis, BMS and MSD. PN has received honoraria in the last 2 years for advisory board membership for AztraZeneca, Esai, BMS, Immunocore, Ipsen, Merck, MSD, Novartis, Pfizer, 4SC. HS has received honoraria for consulting/advisory board membership and speaker's bureau from Novartis, BMS, Sanofi and MSD, along with honoraria for consulting/advisory board membership from Immunocore, Idera, Iovance, Genmab, Genzyme/Regeneron, Macrogenics and Roche. JS received a grant to his institution to fund investigator led research from Immunocore, along with personal fees and institution fees for advisory roles and presentations at meetings from Immunocore. JS also declares a grant for an investigator sponsored trial, personal fees for advisory board attendance and support in attending conferences along with institutional support to run BMS sponsored trials from BMS. JS has received a grant to fund investigator sponsored trials from AstraZeneca, funding from Replimune to his institution to fund Replimune sponsored research and honoraria from Pierre-Fabre. JS has received fees from MSD for advisory board/conference attendance as well as his institution receiving funding to run MSD clinical trials. PL has received personal fees from BMS, Merck, Novartis, GSK, Amgen, Chugai, Pierre-Fabre, NeraCare, Roche and Oncology Education Canada. LT reports research funding from a Sanofi iAward. GH received a research grant from BMS, along with consultancy/advisory relationships with Amgen, Bristol-Myers Squibb, Roche, MSD, Pfizer, Novartis, Sanofi and Pierre Fabre.

## Appendix Supplementary materials

Image, application 1

## References

[1] Wolchok JD, Chiarion-Sileni V, Gonzalez R (2022). Long-term outcomes with nivolumab plus ipilimumab or nivolumab alone versus ipilimumab in patients with advanced melanoma. J Clin Oncol.

[2] Larkin J, Chiarion-Sileni V, Gonzalez R (2019). Five-year survival with combined nivolumab and ipilimumab in advanced melanoma. N Engl J Med.

[3] Hodi FS, Chiarion-Sileni V, Gonzalez R (2018). Nivolumab plus ipilimumab or nivolumab alone versus ipilimumab alone in advanced melanoma (CheckMate 067): 4-year outcomes of a multicentre, randomised, phase 3 trial. Lancet Oncol.

[4] Yarchoan M, Albacker LA, Hopkins AC (2019). PD-L1 expression and tumor mutational burden are independent biomarkers in most cancers. JCI Insight.

[5] Chan TA, Wolchok JD, Snyder A. (2015). Genetic basis for clinical response to CTLA-4 blockade in melanoma. N Engl J Med.

[6] Van Allen EM, Miao D, Schilling B (2015). Genomic correlates of response to CTLA-4 blockade in metastatic melanoma. Science.

[7] Tumeh PC, Harview CL, Yearley JH (2014). PD-1 blockade induces responses by inhibiting adaptive immune resistance. Nature.

[8] Huang AC, Orlowski RJ, Xu X (2019). A single dose of neoadjuvant PD-1 blockade predicts clinical outcomes in resectable melanoma. Nat Med.

[9] Amaria RN, Reddy SM, Tawbi HA (2018). Neoadjuvant immune checkpoint blockade in high-risk resectable melanoma. Nat Med.

[10] Ayers M, Lunceford J, Nebozhyn M (2017). IFN-γ–related mRNA profile predicts clinical response to PD-1 blockade. J Clin Investig.

[11] Jacquelot N, Roberti MP, Enot DP (2017). Predictors of responses to immune checkpoint blockade in advanced melanoma. Nat Commun.

[12] Huang AC, Postow MA, Orlowski RJ (2017). T-cell invigoration to tumour burden ratio associated with anti-PD-1 response. Nature.

[13] Krieg C, Nowicka M, Guglietta S (2018). High-dimensional single-cell analysis predicts response to anti-PD-1 immunotherapy. Nat Med.

[14] Weide B, Martens A, Hassel JC (2016). Baseline Biomarkers for outcome of melanoma patients treated with pembrolizumab. Clin Cancer Res.

[15] Pistillo MP, Fontana V, Morabito A (2019). Soluble CTLA-4 as a favorable predictive biomarker in metastatic melanoma patients treated with ipilimumab: an Italian melanoma intergroup study. Cancer Immunol Immunother.

[16] Ugurel S, Schadendorf D, Horny K (2020). Elevated baseline serum PD-1 or PD-L1 predicts poor outcome of PD-1 inhibition therapy in metastatic melanoma. Ann Oncol.

[17] Laino AS, Woods D, Vassallo M (2020). Serum interleukin-6 and C-reactive protein are associated with survival in melanoma patients receiving immune checkpoint inhibition. J Immunother Cancer.

[18] Sanmamed MF, Perez-Gracia JL, Schalper KA (2017). Changes in serum interleukin-8 (IL-8) levels reflect and predict response to anti-PD-1 treatment in melanoma and non-small-cell lung cancer patients. Ann Oncol.

[19] Briukhovetska D, Dörr J, Endres S, Libby P, Dinarello CA, Kobold S. (2021). Interleukins in cancer: from biology to therapy. Nat Rev Cancer.

[20] Schwartz LH, Litière S, de Vries E (2016). RECIST 1.1-update and clarification: from the RECIST committee. Eur J Cancer.

[21] Assarsson E, Lundberg M, Holmquist G (2014). Homogenous 96-plex PEA immunoassay exhibiting high sensitivity, specificity, and excellent scalability. PLoS One.

[22] Li J, Ji L (2005). Adjusting multiple testing in multilocus analyses using the eigenvalues of a correlation matrix. Heredity.

[23] Blackford JU, Salomon RM, Waller NG. (2009). Detecting change in biological rhythms: a multivariate permutation test approach to Fourier-transformed data. Chronobiol Int.

[24] Hoejberg L, Bastholt L, Schmidt H. (2012). Interleukin-6 and melanoma. Melanoma Res.

[25] Yamazaki N, Kiyohara Y, Uhara H (2017). Cytokine biomarkers to predict antitumor responses to nivolumab suggested in a phase 2 study for advanced melanoma. Cancer Sci.

[26] Czyz M. (2018). HGF/c-MET signaling in melanocytes and melanoma. Int J Mol Sci.

[27] Gaffal E, Landsberg J, Bald T, Sporleder A, Kohlmeyer J, Tüting T. (2011). Neonatal UVB exposure accelerates melanoma growth and enhances distant metastases in Hgf-Cdk4(R24C) C57BL/6 mice. Int J Cancer.

[28] Kubo Y, Fukushima S, Inamori Y (2019). Serum concentrations of HGF are correlated with response to anti-PD-1 antibody therapy in patients with metastatic melanoma. J Dermatol Sci.

[29] Debes GF, Diehl MC. (2011). CCL8 and skin T cells–an allergic attraction. Nat Immunol.

[30] Chen XJ, Deng YR, Wang ZC (2019). Hypoxia-induced ZEB1 promotes cervical cancer progression via CCL8-dependent tumour-associated macrophage recruitment. Cell Death Dis.

[31] Zhang X, Chen L, Dang WQ (2020). CCL8 secreted by tumor-associated macrophages promotes invasion and stemness of glioblastoma cells via ERK1/2 signaling. Lab Invest.

[32] Hugo W, Zaretsky JM, Sun L (2016). Genomic and transcriptomic features of response to anti-PD-1 therapy in metastatic melanoma. Cell.

[33] Akbaraly TN, Hamer M, Ferrie JE (2013). Chronic inflammation as a determinant of future aging phenotypes. CMAJ.

[34] Mehmi I, Hamid O, Hodi FS (2021). Ipilimumab, nivolumab and tocilizumab as first-line therapy for advanced melanoma. J Clin Oncol.

[35] Chow MT, Ozga AJ, Servis RL (2019). Intratumoral activity of the CXCR3 chemokine system is required for the efficacy of anti-PD-1 therapy. Immunity.

[36] Bridge JA, Lee JC, Daud A, Wells JW, Bluestone JA. (2018). Cytokines, chemokines, and other biomarkers of response for checkpoint inhibitor therapy in skin cancer. Front Med.

[37] Conforti C, Zalaudek I. (2021). Epidemiology and risk factors of melanoma: a review. Dermatol Pract Concept.

[38] Saginala K, Barsouk A, Aluru JS, Rawla P, Barsouk A. (2021). Epidemiology of melanoma. Med Sci (Basel).

[39] Rastrelli M, Tropea S, Rossi CR, Alaibac M. Melanoma: epidemiology, risk factors, pathogenesis, diagnosis and classification. In Vivo. 2014;28(6):1005–11. 25398793

[40] Rigel DS. (2010). Epidemiology of melanoma. Semin Cutan Med Surg.

[41] Coudriet GM, He J, Trucco M, Mars WM, Piganelli JD. (2010). Hepatocyte growth factor modulates interleukin-6 production in bone marrow derived macrophages: implications for inflammatory mediated diseases. PLoS One.

