# Supplementary material for: Circulating inflammatory proteins associate with response to immune checkpoint inhibition therapy in patients with advanced melanoma
Source: eBioMedicine. 2022 Aug 22;83:104235. doi: 10.1016/j.ebiom.2022.104235 (PMC9421308; doi:10.1016/j.ebiom.2022.104235)
Supplement: Supplementary file 1 [file mmc1.pdf]

Supplementary material for

Circulating inflammatory proteins associate with response to immune checkpoint inhibition therapy in patients with advanced melanoma

Niccolò Rossi PhD[1*], Karla A. Lee MD[1*], Maria V. Bermudez BSc.[2], Alessia Visconti PhD[1], Andrew Maltez Thomas PhD[3], Laura A. Bolte BSc[4], Johannes R. Björk PhD[4], Laura Kist de Ruijter MD[5], Julia Newton-Bishop MB MD[6], Mark Harland PhD[6], Heather M. Shaw MD PhD[7], Mark Harries MD PhD[8], Joseph Sacco MD PhD[9], Ruth Board MD PhD[10], Paul Lorigan MD[11,12], Elisabeth G. E. de Vries MD PhD[5], Nicola Segata PhD[3], Leonie S. Taams PhD[2], Sophie Papa MD PhD[13,8], Tim D. Spector MB MD[1], Paul Nathan MD PhD[7], Rinse K. Weersma MD PhD[4], Geke A. P. Hospers MD PhD[5], Rudolf S.N. Fehrmann MD PhD[5], Veronique Bataille MD PhD[1,7§], Mario Falchi PhD[1§]

1. Department of Twin Research and Genetic Epidemiology, King's College London, U.K.
2. Centre for Inflammation Biology and Cancer Immunology, King's College London, U.K.
3. Department CIBIO, University of Trento, Trento, Italy.
4. Department of Gastroenterology and Hepatology University of Groningen and University Medical Center Groningen, the Netherlands
5. Department of Medical Oncology, University of Groningen, University Medical Center Groningen, the Netherlands
6. Division of Haematology and Immunology, Institute of Medical Research at St James's, University of Leeds, U.K.
7. Department of Medical Oncology, Mount Vernon Cancer Centre, Northwood, U.K.
8. Department of Medical Oncology, Guy's and St Thomas' NHS Foundation Trust, London, U.K.
9. Liverpool Clatterbridge Cancer Centre, Liverpool, U.K.
10. Department of Oncology, Lancashire Teaching Hospitals NHS Trust, Preston, U.K.
11. The Christie NHS Foundation Trust, Manchester, U.K.
12. Division of Cancer Sciences, University of Manchester, U.K.
13. School of Cancer and Pharmaceutical Studies, King's College London, U.K.

*Equal contribution
§Shared senior authorship

Corresponding authors e-mail:mario.falchi@kcl.ac.uk veronique.bataille@kcl.ac.uk

Supplementary Methods

Study patients

PRIMM-UK (NCT03643289) is a prospective observational study recruiting patients across the UK. PRIMM-UK was sponsored by East & North Hertfordshire NHS Trust with an ethical approval from the South Central Berkshire committee of the Research Ethics Services (RES) of the NHS. LEEDS was recruited within a retrospective cohort study managed by the University of Leeds and named "Developing a Blood Test of Immunity in Illness: A study examining the peripheral blood transcriptome in patients with cancer, autoimmune disease, immunodeficiency or iatrogenic immune suppression" (REC Ref: 15/NW/0933). COLIPI (Identification of Predictive Parameters for Colitis in Melanoma Patients Treated With Immunotherapy), POINTING (towards patient -tailored cancer immunotherapy supported by a multifaceted predictive signature composed of integrative omics and molecular imaging), and OncoLifeS (Oncological Life Study) are three observational studies prospectively recruiting patients with melanoma from the Dutch population. OncoLifeS (METc number 2010/109), COLIPI (METc number 2012/085, NCT02600143) and POINTING (METc number 2018/350, NCT04193956) have all been approved by the Medical Ethical Committee (in Dutch: Medisch Ethische Toetsingsingscommissie or METc) of the University Medical Center Groningen (UMCG) in the Netherlands. OncoLifeS information is available on the Netherlands Trial Register: NTR: https://www.trialregister.nl/trial/7839. Written informed consent was obtained from all patients.

Clinical evaluation

Collected clinical/demographic data used in this study include age at ICI initiation, sex, body mass index (BMI), BRAF mutation status, metastatic stage, lactate dehydrogenase (LDH) levels, Eastern Cooperative Oncology Group performance-status (ECOG-PS), and prescribed ICI regimen. LDH levels were dichotomized according to the upper limit of normal for each centre. Radiological evaluation, consisting of computed tomography (CT) scan of the chest, abdomen and pelvis and MRI of the brain, was performed before starting immunotherapy. A small number of patients underwent a combination of positron emission tomography (PET) and CT scan (PET-CT). Follow-up radiological evaluation was performed every 10–14 weeks as long as the patient received ICI therapy. Additional CT- and/or MRI scans were performed when there was suspicion of progression. If the first radiological evaluation after the start of treatment was inconclusive, a confirmatory scan was performed 4-12 weeks later.

Sample collection

Venous blood samples were taken at the respective hospitals attended by the patients. Serum was obtained by centrifugation and stored at each site at −80° C until shipping to King's College London, where they were aliquoted for analysis. For each serum sample, 40 µL was shipped by courier on dry ice to the clinical biomarkers' facility OLINK at SciLifeLab (Uppsala, Sweden) in randomised 96-well plates for proteomic characterisation.

Luminex assay

Validation of serum protein expression levels was performed using a custom Luminex assay (R&D systems, MN, USA). Serum samples (n=81) were centrifuged at 16,000 x g for 4 minutes, then diluted two-fold and plated in duplicate. The assay was performed per manufacturer's guidelines and acquired with a Luminex Flexmap3D. Protein concentrations were calculated using a standard curve for mean fluorescence intensity of each analyte against picograms/millilitre (pg/mL). Measures below or above the assay limits of detection (n=3, corresponding to 0·6% of the tested samples) were excluded. Samples showing extreme variation between duplicate measures (*i.e.*, >4 SD from the protein sample mean) were removed ($n_{IL\text{-}6}$=1, n_{HGF}=2, and $n_{MCP\text{-}2}$=2), and concentrations from duplicate wells were averaged. Outliers, defined as those values deviating by more than four SD from the protein sample mean, were removed.

Multivariate permutation

At each permutation iteration, proteins were randomly permuted as a single set, thereby preserving their natural correlations. We assessed the absolute test statistics (*i.e.*, absolute z-score) for each protein in each random dataset i (Z_i), and used a step-down procedure to assess the significance of each association observed in the original dataset. The largest absolute test statistics from each permutation ($Z_{i\ max}$) was used to define the distribution of the test statistics under the null hypothesis (Z_0). We selected the largest absolute test statistics across all observed associations (Z_{max}), and estimated an empirical p-value (p_{emp}) as the probability of observing a z-score Z_0 as or more extreme than the observed one (Z_{max}). If p_{emp} was $<0{\cdot}05$, we considered the association significant, and its test statistics were removed from both the original and the random datasets. We repeated this procedure until p_{emp} became $>0{\cdot}05$, and considered all remaining associations not significant.[1]

Table S1. Meta-analysis of the association between ORR and pre-treatment levels of 77 serum inflammatory proteins in 48 patients with advanced melanoma from the UK and 39 patients from the NL. Protein differences between responders and non-responders were supplied to a random-effects meta-analysis model using the restricted maximum-likelihood estimator. For each study centre, we report the mean difference (MD) of log_2-scaled protein levels between responders (R) and non-responders (NR), and sampling variance (σ^2). Meta-analysis statistics are shown, including the mean log_2-FC (Summary effect), along with its lower (Lower) and upper (Upper) 95% CI bounds, p-value (p), and Bonferroni-corrected p-value (p_{adj}), and the Cochran's Q test of heterogeneity statistics (QE) and p-value (p_{QE}).

	UK				NL										
**Protein**	**R**	**NR**	**MD**	**σ^2**	**R**	**NR**	**MD**	**σ^2**	**Summary effect**	**Lower**	**Upper**	**p**	**p_{adj}**	**QE**	**p_{QE}**
HGF	23	24	0·41	0·02	23	16	0·41	0·02	0·41	0·19	0·63	2·29x10^{-4}	9·62x10^{-3}	0·00	1·00x10^{0}
IL-6	23	25	0·96	0·10	23	16	0·79	0·18	0·90	0·41	1·39	3·31x10^{-4}	1·39x10^{-2}	0·10	7·56x10^{-1}
MCP-2	23	25	0·53	0·04	23	16	0·43	0·04	0·48	0·19	0·76	1·02x10^{-3}	4·28x10^{-2}	0·11	7·41x10^{-1}
IL-18R1	23	25	0·33	0·02	23	16	0·29	0·02	0·32	0·12	0·51	1·56x10^{-3}	>0·05	0·04	8·33x10^{-1}
CCL4	23	25	0·34	0·04	23	16	0·47	0·03	0·41	0·15	0·67	2·11x10^{-3}	>0·05	0·22	6·36x10^{-1}
CSF-1	23	25	0·10	0·00	23	16	0·20	0·00	0·15	0·05	0·24	2·28x10^{-3}	>0·05	1·01	3·15x10^{-1}
IL-17A	22	23	0·30	0·02	23	14	0·57	0·06	0·37	0·12	0·62	3·36x10^{-3}	>0·05	0·95	3·30x10^{-1}
TNF	23	25	0·55	0·04	23	16	0·26	0·05	0·42	0·12	0·72	6·04x10^{-3}	>0·05	0·91	3·41x10^{-1}
CCL20	23	25	0·42	0·09	23	15	0·60	0·07	0·53	0·15	0·91	6·83x10^{-3}	>0·05	0·22	6·38x10^{-1}
PD-L1	23	25	0·19	0·02	23	16	0·27	0·01	0·23	0·06	0·41	7·21x10^{-3}	>0·05	0·19	6·61x10^{-1}
FGF-21	23	25	0·47	0·13	23	16	1·01	0·16	0·71	0·18	1·24	8·36x10^{-3}	>0·05	1·02	3·13x10^{-1}
LIF-R	23	25	0·11	0·01	23	16	0·20	0·01	0·16	0·04	0·28	8·57x10^{-3}	>0·05	0·50	4·79x10^{-1}
CDCP1	23	25	0·33	0·03	23	16	0·36	0·04	0·34	0·09	0·60	8·93x10^{-3}	>0·05	0·01	9·15x10^{-1}
OSM	23	25	0·57	0·09	23	16	0·51	0·08	0·54	0·14	0·95	9·09x10^{-3}	>0·05	0·02	8·85x10^{-1}
CCL3	23	24	0·44	0·08	23	16	0·47	0·06	0·46	0·10	0·82	1·31x10^{-2}	>0·05	0·01	9·22x10^{-1}
CXCL11	23	25	0·63	0·08	23	16	0·35	0·10	0·51	0·09	0·92	1·77x10^{-2}	>0·05	0·44	5·07x10^{-1}
LAP (TGFB1)	23	25	0·19	0·02	23	16	0·30	0·02	0·24	0·04	0·44	1·94x10^{-2}	>0·05	0·30	5·81x10^{-1}
uPA	23	25	0·18	0·01	23	16	0·14	0·01	0·15	0·02	0·28	2·09x10^{-2}	>0·05	0·12	7·29x10^{-1}
CXCL1	23	25	0·24	0·02	23	16	0·28	0·03	0·26	0·04	0·48	2·27x10^{-2}	>0·05	0·02	8·86x10^{-1}
IL-8	23	25	0·65	0·10	23	15	0·38	0·12	0·52	0·07	0·98	2·49x10^{-2}	>0·05	0·33	5·63x10^{-1}
ADA	23	25	0·21	0·01	23	16	0·24	0·03	0·22	0·02	0·41	2·68x10^{-2}	>0·05	0·02	8·78x10^{-1}
CCL23	23	25	0·24	0·02	23	16	0·28	0·03	0·25	0·03	0·48	2·85x10^{-2}	>0·05	0·03	8·53x10^{-1}
TNFRSF9	23	25	0·17	0·02	23	16	0·24	0·02	0·21	0·01	0·41	3·77x10^{-2}	>0·05	0·15	7·02x10^{-1}
VEGFA	23	25	0·27	0·03	23	16	0·26	0·04	0·27	0·01	0·52	4·09x10^{-2}	>0·05	0·00	9·76x10^{-1}
CD40	23	25	0·14	0·01	23	16	0·22	0·02	0·17	0·01	0·34	4·14x10^{-2}	>0·05	0·19	6·59x10^{-1}
IL-18	23	25	0·25	0·03	23	16	0·68	0·07	0·42	0·00	0·83	4·77x10^{-2}	>0·05	1·83	1·76x10^{-1}
IL-7	23	25	0·34	0·03	23	16	0·16	0·03	0·25	0·00	0·50	5·07x10^{-2}	>0·05	0·49	4·82x10^{-1}
OPG	23	25	0·15	0·02	23	16	0·24	0·02	0·19	-0·01	0·38	5·74x10^{-2}	>0·05	0·22	6·42x10^{-1}
MCP-3	23	25	0·46	0·06	23	15	0·18	0·04	0·29	-0·02	0·59	6·67x10^{-2}	>0·05	0·78	3·78x10^{-1}
IL-10	23	25	0·31	0·04	23	16	0·26	0·12	0·30	-0·02	0·62	6·76x10^{-2}	>0·05	0·02	8·82x10^{-1}
CXCL10	23	25	0·45	0·07	23	16	0·26	0·11	0·37	-0·04	0·79	7·75x10^{-2}	>0·05	0·21	6·50x10^{-1}
SCF	23	25	-0·18	0·03	23	16	-0·37	0·05	-0·25	-0·53	0·03	7·76x10^{-2}	>0·05	0·40	5·28x10^{-1}
IL-15RA	23	25	0·05	0·01	23	16	0·21	0·01	0·13	-0·03	0·29	1·02x10^{-1}	>0·05	1·58	2·09x10^{-1}
SLAMF1	23	25	0·06	0·02	23	16	0·19	0·01	0·13	-0·03	0·30	1·11x10^{-1}	>0·05	0·55	4·58x10^{-1}
CD8A	23	25	0·29	0·05	23	16	0·23	0·07	0·27	-0·07	0·60	1·16x10^{-1}	>0·05	0·03	8·64x10^{-1}
CD5	23	25	0·10	0·02	23	16	0·14	0·01	0·12	-0·04	0·29	1·46x10^{-1}	>0·05	0·04	8·40x10^{-1}
IL-12B	23	25	0·27	0·04	23	16	0·14	0·08	0·23	-0·09	0·56	1·60x10^{-1}	>0·05	0·14	7·13x10^{-1}
TNFSF14	23	25	0·21	0·07	23	16	0·27	0·06	0·25	-0·11	0·60	1·70x10^{-1}	>0·05	0·03	8·72x10^{-1}
CXCL6	23	25	0·08	0·03	23	16	0·29	0·04	0·17	-0·07	0·42	1·70x10^{-1}	>0·05	0·68	4·09x10^{-1}

GDNF	23	25	0·13	0·02	22	16	0·10	0·03	0·12	-0·08	0·33	2·38x10^{-1}	>0·05	0·02	8·88x10^{-1}
TRANCE	23	25	-0·21	0·06	23	16	-0·23	0·09	-0·22	-0·59	0·16	2·54x10^{-1}	>0·05	0·00	9·51x10^{-1}
IFN-γ	23	24	0·23	0·09	23	16	0·84	0·57	0·32	-0·24	0·88	2·66x10^{-1}	>0·05	0·57	4·49x10^{-1}
AXIN1	23	25	-0·07	0·10	23	16	0·30	0·04	0·19	-0·16	0·53	2·91x10^{-1}	>0·05	0·90	3·44x10^{-1}
IL-17C	22	25	0·13	0·05	23	16	0·19	0·05	0·16	-0·15	0·47	3·10x10^{-1}	>0·05	0·03	8·63x10^{-1}
CXCL9	23	25	0·29	0·08	23	16	0·10	0·16	0·23	-0·22	0·67	3·18x10^{-1}	>0·05	0·16	6·93x10^{-1}
IL-20RA	22	20	0·01	0·01	21	16	0·23	0·02	0·11	-0·10	0·32	3·20x10^{-1}	>0·05	1·85	1·74x10^{-1}
NT-3	23	24	0·16	0·02	23	16	0·01	0·02	0·09	-0·09	0·26	3·28x10^{-1}	>0·05	0·66	4·16x10^{-1}
CD244	23	25	0·06	0·01	23	16	0·10	0·01	0·08	-0·08	0·24	3·45x10^{-1}	>0·05	0·05	8·28x10^{-1}
DNER	23	25	0·08	0·02	23	16	0·08	0·02	0·08	-0·10	0·25	3·78x10^{-1}	>0·05	0·00	9·91x10^{-1}
MMP-10	23	24	0·24	0·04	23	16	-0·02	0·04	0·12	-0·16	0·40	4·10x10^{-1}	>0·05	0·86	3·54x10^{-1}
IL-10RB	23	24	-0·02	0·01	23	16	0·14	0·01	0·06	-0·10	0·22	4·35x10^{-1}	>0·05	1·89	1·69x10^{-1}
FGF-19	23	25	0·02	0·05	23	16	-0·41	0·08	-0·16	-0·58	0·26	4·60x10^{-1}	>0·05	1·46	2·26x10^{-1}
MCP-1	23	25	-0·07	0·03	23	16	0·47	0·03	0·20	-0·33	0·73	4·61x10^{-1}	>0·05	5·38	2·03x10^{-2}
TGF-α	23	24	0·20	0·07	23	16	0·08	0·05	0·13	-0·22	0·47	4·63x10^{-1}	>0·05	0·11	7·41x10^{-1}
CXCL5	23	25	-0·04	0·06	23	16	-0·23	0·07	-0·13	-0·48	0·22	4·68x10^{-1}	>0·05	0·31	5·79x10^{-1}
SIRT2	23	25	0·14	0·07	23	16	0·11	0·06	0·12	-0·23	0·48	4·96x10^{-1}	>0·05	0·01	9·35x10^{-1}
IL-10RA	23	23	0·31	0·05	20	16	-0·01	0·02	0·11	-0·20	0·41	5·00x10^{-1}	>0·05	1·67	1·97x10^{-1}
CCL19	23	25	-0·01	0·05	23	16	0·50	0·13	0·16	-0·31	0·62	5·05x10^{-1}	>0·05	1·41	2·35x10^{-1}
TNFB	23	25	-0·01	0·02	23	16	0·16	0·02	0·07	-0·13	0·26	5·16x10^{-1}	>0·05	0·64	4·24x10^{-1}
ST1A1	23	23	-0·18	0·14	23	16	0·61	0·10	0·24	-0·53	1·01	5·39x10^{-1}	>0·05	2·51	1·13x10^{-1}
STAMBP	23	25	0·09	0·04	23	16	0·06	0·03	0·07	-0·19	0·33	5·97x10^{-1}	>0·05	0·01	9·14x10^{-1}
CCL25	23	25	-0·14	0·04	23	16	-0·02	0·02	-0·06	-0·29	0·17	5·98x10^{-1}	>0·05	0·26	6·08x10^{-1}
EN-RAGE	23	25	0·09	0·11	23	16	0·11	0·06	0·10	-0·29	0·49	6·11x10^{-1}	>0·05	0·00	9·59x10^{-1}
CCL28	23	25	0·25	0·01	23	16	-0·08	0·01	0·08	-0·24	0·41	6·22x10^{-1}	>0·05	3·98	4·61x10^{-2}
TRAIL	23	25	0·08	0·01	23	16	-0·01	0·01	0·03	-0·10	0·17	6·40x10^{-1}	>0·05	0·49	4·85x10^{-1}
MCP-4	23	25	-0·16	0·05	23	16	0·01	0·05	-0·07	-0·38	0·24	6·50x10^{-1}	>0·05	0·28	5·95x10^{-1}
CASP-8	23	25	-0·01	0·05	23	16	0·24	0·09	0·08	-0·27	0·43	6·59x10^{-1}	>0·05	0·44	5·06x10^{-1}
CCL11	23	25	0·09	0·03	23	16	0·01	0·02	0·04	-0·16	0·25	6·64x10^{-1}	>0·05	0·14	7·10x10^{-1}
TWEAK	23	25	0·02	0·01	23	16	0·06	0·02	0·04	-0·13	0·21	6·73x10^{-1}	>0·05	0·05	8·23x10^{-1}
CX3CL1	23	25	0·05	0·01	23	16	0·02	0·02	0·03	-0·14	0·21	7·03x10^{-1}	>0·05	0·03	8·63x10^{-1}
CST5	23	25	0·00	0·03	23	16	-0·07	0·02	-0·04	-0·27	0·18	7·05x10^{-1}	>0·05	0·09	7·60x10^{-1}
MMP-1	23	25	0·04	0·04	22	16	0·07	0·05	0·05	-0·24	0·35	7·30x10^{-1}	>0·05	0·01	9·24x10^{-1}
FGF-23	22	23	-0·08	0·02	22	16	0·17	0·03	0·03	-0·21	0·28	7·81x10^{-1}	>0·05	1·27	2·59x10^{-1}
CD6	23	25	0·07	0·03	23	16	0·00	0·03	0·03	-0·22	0·28	8·16x10^{-1}	>0·05	0·09	7·70x10^{-1}
FGF-5	23	24	0·10	0·01	23	16	-0·13	0·01	0·00	-0·22	0·22	9·81x10^{-1}	>0·05	2·26	1·32x10^{-1}
4E-BP1	23	25	0·36	0·15	23	16	-0·35	0·15	0·01	-0·69	0·70	9·86x10^{-1}	>0·05	1·69	1·94x10^{-1}
Flt3L	23	25	-0·13	0·02	23	16	0·19	0·04	0·00	-0·31	0·31	9·93x10^{-1}	>0·05	1·70	1·93x10^{-1}

Table S2. Association between ORR and pre-treatment levels of IL-6, HGF, and MCP-2 measured through Luminex. Protein differences between 41 responders and 40 non-responders were assessed using Wilcoxon's test ($p_{Wilcoxon}$). Median, lower (Q1) and upper (Q3) quartiles of protein levels (pg/mL) among responders and non-responders are shown. Odds ratios (ORs) and p-values ($p_{regression}$) were estimated using logistic regression, while 95% CIs were estimated via bootstrapping. Age, sex, BMI, and metastatic stage were included as covariates in the model.

	**Responders**				**Non-Responders**								
**Protein**	**N**	**median**	**Q1**	**Q3**	**N**	**median**	**Q1**	**Q3**	**mean FC**	**$p_{Wilcoxon}$**	**OR**	**95% CI**	**$p_{regression}$**
IL-6	41	1·14	0·79	1·83	39	1·71	1·28	3·81	2·46	$1{\cdot}44 \times 10^{-3}$	0·76	0·32-0·90	$7{\cdot}70 \times 10^{-3}$
HGF	41	170·03	132·13	213·32	37	224·44	171·67	294·58	1·36	$8{\cdot}75 \times 10^{-3}$	0·99	0·97-0·99	$2{\cdot}78 \times 10^{-3}$
MCP-2	39	47·41	34·37	59·50	39	59·52	48·52	79·77	1·32	$2{\cdot}15 \times 10^{-3}$	0·97	0·93-1·00	$3{\cdot}85 \times 10^{-2}$

Table S3. Protein changes from baseline at early (2-5 weeks after ICI initiation) and late (5-12 weeks after ICI initiation) follow-up visits. Protein changes were assessed on Olink data in 46 and 35 patients with advanced melanoma at early and late follow-up time windows, respectively, using a two-sided Wilcoxon test for paired data (p). Median change of log_2-scaled protein levels, along with the lower (Q1) and upper (Q3) quartiles, are shown. A Wilcoxon empirical p-value (p_{emp}) was determined using multivariate permutation.

	**Early follow-up window**						**Late follow-up window**					
**Protein**	**N**	**median**	**Q1**	**Q3**	**p**	**p_{emp}**	**N**	**median**	**Q1**	**Q3**	**p**	**p_{emp}**
TNFRSF9	45	0·49	0·25	0·77	1·57x10^{-10}	2·84x10^{-2}	35	0·45	0·18	0·68	3·34x10^{-7}	>0·05
CXCL9	46	1·09	0·39	1·90	5·81x10^{-8}	4·12x10^{-2}	35	1·31	0·53	1·81	5·26x10^{-8}	4·04x10^{-2}
IL-10	46	0·63	0·20	1·13	2·93x10^{-7}	>0·05	35	0·55	0·14	0·91	2·87x10^{-5}	>0·05
CXCL11	46	0·69	0·23	1·31	5·10x10^{-7}	>0·05	35	0·45	0·19	1·31	1·61x10^{-5}	>0·05
CCL19	46	0·46	0·20	1·12	5·51x10^{-7}	>0·05	35	0·21	-0·23	0·91	1·31x10^{-1}	>0·05
CD5	46	0·28	0·06	0·52	1·26x10^{-6}	>0·05	35	0·34	0·09	0·60	5·05x10^{-7}	>0·05
IL-12B	46	0·41	0·13	0·74	1·02x10^{-5}	>0·05	35	0·35	0·10	0·87	6·65x10^{-3}	>0·05
CXCL10	46	0·77	0·20	1·50	1·23x10^{-5}	>0·05	35	0·71	0·19	1·50	5·45x10^{-5}	>0·05
MCP-3	46	0·38	0·07	0·74	2·26x10^{-5}	>0·05	35	0·12	-0·25	0·73	8·10x10^{-2}	>0·05
IL-15RA	45	0·19	0·01	0·33	2·68x10^{-5}	>0·05	35	0·20	0·06	0·39	9·74x10^{-7}	>0·05
TNFB	46	0·25	0·04	0·36	7·43x10^{-5}	>0·05	35	0·24	0·01	0·39	5·97x10^{-3}	>0·05
IL-17A	44	0·36	0·07	0·63	8·18x10^{-5}	>0·05	33	0·31	-0·12	0·70	1·44x10^{-2}	>0·05
IFN-γ	46	0·95	0·11	1·77	9·71x10^{-5}	>0·05	35	0·49	-0·78	0·89	4·13x10^{-1}	>0·05
OSM	46	0·48	0·14	0·88	1·47x10^{-4}	>0·05	35	0·12	-0·59	0·77	2·73x10^{-1}	>0·05
PD-L1	46	0·22	-0·06	0·48	1·99x10^{-4}	>0·05	35	0·16	-0·04	0·39	2·63x10^{-2}	>0·05
Flt3L	46	0·19	0·01	0·49	2·68x10^{-4}	>0·05	35	0·14	-0·33	0·42	5·65x10^{-1}	>0·05
CXCL1	46	0·19	0·00	0·35	3·10x10^{-4}	>0·05	35	0·12	-0·17	0·35	1·27x10^{-1}	>0·05
TNF	45	0·29	-0·02	0·75	4·06x10^{-4}	>0·05	35	0·18	-0·14	0·65	1·91x10^{-2}	>0·05
IL-10RB	44	0·13	-0·01	0·20	8·58x10^{-4}	>0·05	34	0·08	-0·02	0·20	1·31x10^{-2}	>0·05
CD6	46	0·25	-0·07	0·61	1·10x10^{-3}	>0·05	35	0·45	0·07	0·61	1·09x10^{-4}	>0·05
FGF-23	41	0·14	0·01	0·34	1·32x10^{-3}	>0·05	31	-0·07	-0·23	0·30	8·85x10^{-1}	>0·05
CCL3	45	0·31	-0·02	0·85	1·60x10^{-3}	>0·05	35	0·20	-0·34	0·62	1·95x10^{-1}	>0·05
TNFSF14	46	0·29	-0·05	0·87	1·75x10^{-3}	>0·05	35	0·09	-0·61	0·62	6·68x10^{-1}	>0·05
HGF	45	0·14	-0·03	0·40	2·14x10^{-3}	>0·05	35	0·08	-0·21	0·40	6·25x10^{-2}	>0·05
IL-18	46	0·25	-0·03	0·60	2·41x10^{-3}	>0·05	35	0·31	-0·09	0·60	5·65x10^{-3}	>0·05
TRAIL	46	0·11	-0·05	0·31	2·51x10^{-3}	>0·05	35	0·17	-0·05	0·25	6·74x10^{-2}	>0·05
CXCL6	46	0·16	-0·05	0·39	2·61x10^{-3}	>0·05	35	0·09	-0·13	0·27	1·31x10^{-1}	>0·05
CSF-1	46	0·08	-0·03	0·19	2·71x10^{-3}	>0·05	35	0·05	-0·08	0·19	1·27x10^{-1}	>0·05
CCL4	45	0·18	-0·04	0·48	2·97x10^{-3}	>0·05	35	-0·01	-0·20	0·25	9·29x10^{-1}	>0·05
CD8A	46	0·21	-0·12	0·41	3·97x10^{-3}	>0·05	35	0·14	-0·14	0·56	1·37x10^{-2}	>0·05
CCL23	46	0·15	-0·12	0·49	5·94x10^{-3}	>0·05	35	0·08	-0·02	0·52	3·80x10^{-3}	>0·05
CD40	45	0·13	-0·02	0·41	8·51x10^{-3}	>0·05	35	0·12	-0·03	0·46	5·34x10^{-3}	>0·05
SLAMF1	46	0·11	-0·08	0·38	9·33x10^{-3}	>0·05	35	0·08	-0·22	0·30	2·65x10^{-1}	>0·05
uPA	46	0·14	-0·05	0·29	1·22x10^{-2}	>0·05	35	0·07	-0·15	0·24	1·79x10^{-1}	>0·05
CXCL5	46	0·12	-0·08	0·36	1·26x10^{-2}	>0·05	34	0·13	0·04	0·26	5·01x10^{-4}	>0·05
TGF-α	46	0·27	-0·09	0·66	1·62x10^{-2}	>0·05	35	-0·04	-0·52	0·61	8·27x10^{-1}	>0·05
CDCP1	45	0·24	-0·12	0·55	1·67x10^{-2}	>0·05	34	0·23	-0·09	0·45	1·25x10^{-2}	>0·05
MCP-4	46	0·14	-0·12	0·44	2·41x10^{-2}	>0·05	35	0·02	-0·27	0·24	7·90x10^{-1}	>0·05
IL-17C	44	0·14	-0·08	0·42	2·94x10^{-2}	>0·05	35	0·22	-0·24	0·69	1·90x10^{-1}	>0·05
VEGFA	46	0·11	-0·08	0·37	2·96x10^{-2}	>0·05	35	0·00	-0·18	0·29	4·81x10^{-1}	>0·05

CCL20	46	0·20	-0·35	0·95	$3{\cdot}41x10^{-2}$	>0·05	35	0·32	-0·45	0·95	$9{\cdot}02x10^{-2}$	>0·05
FGF-21	45	-0·39	-0·84	0·33	$4{\cdot}02x10^{-2}$	>0·05	35	-0·49	-0·84	0·32	$6{\cdot}01x10^{-2}$	>0·05
CD244	46	0·09	-0·08	0·23	$5{\cdot}68x10^{-2}$	>0·05	35	0·05	-0·15	0·23	$3{\cdot}85x10^{-1}$	>0·05
STAMBP	46	0·27	-0·25	0·80	$5{\cdot}68x10^{-2}$	>0·05	34	0·02	-0·41	0·44	1·00E+00	>0·05
MCP-1	46	0·11	-0·09	0·32	$6{\cdot}13x10^{-2}$	>0·05	35	-0·11	-0·43	0·30	$2{\cdot}07x10^{-1}$	>0·05
ST1A1	45	0·34	-0·61	1·55	$6{\cdot}21x10^{-2}$	>0·05	35	-0·17	-0·64	0·49	$3{\cdot}85x10^{-1}$	>0·05
AXIN1	46	0·27	-0·40	1·19	$7{\cdot}30x10^{-2}$	>0·05	35	0·00	-0·73	0·40	$3{\cdot}77x10^{-1}$	>0·05
IL-18R1	45	0·11	-0·13	0·30	$8{\cdot}21x10^{-2}$	>0·05	34	0·09	-0·15	0·22	$2{\cdot}78x10^{-1}$	>0·05
SIRT2	46	0·28	-0·64	1·29	$8{\cdot}64x10^{-2}$	>0·05	34	0·12	-0·50	0·74	$4{\cdot}36x10^{-1}$	>0·05
MCP-2	45	0·05	-0·04	0·29	$9{\cdot}06x10^{-2}$	>0·05	34	-0·12	-0·32	0·11	$9{\cdot}07x10^{-2}$	>0·05
MMP-1	43	0·06	-0·12	0·21	$1{\cdot}09x10^{-1}$	>0·05	34	-0·05	-0·17	0·09	$2{\cdot}09x10^{-1}$	>0·05
SCF	45	-0·05	-0·28	0·09	$1{\cdot}12x10^{-1}$	>0·05	34	-0·06	-0·34	0·24	$4{\cdot}99x10^{-1}$	>0·05
ADA	45	0·06	-0·15	0·34	$1{\cdot}31x10^{-1}$	>0·05	34	0·13	-0·08	0·40	$7{\cdot}69x10^{-3}$	>0·05
CASP-8	46	0·11	-0·44	0·86	$1{\cdot}57x10^{-1}$	>0·05	35	0·15	-0·40	0·68	$3{\cdot}77x10^{-1}$	>0·05
IL-20RA	40	0·08	-0·10	0·17	$1{\cdot}70x10^{-1}$	>0·05	31	-0·03	-0·19	0·17	$4{\cdot}68x10^{-1}$	>0·05
LAP (TGFB1)	46	0·05	-0·18	0·37	$1{\cdot}72x10^{-1}$	>0·05	35	0·08	-0·13	0·27	$2{\cdot}13x10^{-1}$	>0·05
TRANCE	46	0·08	-0·17	0·50	$1{\cdot}74x10^{-1}$	>0·05	35	0·37	-0·16	0·55	$3{\cdot}13x10^{-2}$	>0·05
IL-6	45	0·13	-0·20	0·44	$1{\cdot}77x10^{-1}$	>0·05	35	-0·14	-0·91	0·33	$1{\cdot}58x10^{-1}$	>0·05
CST5	46	0·04	-0·15	0·25	$1{\cdot}99x10^{-1}$	>0·05	35	0·08	-0·11	0·23	$3{\cdot}59x10^{-1}$	>0·05
CCL28	46	0·04	-0·16	0·29	$2{\cdot}28x10^{-1}$	>0·05	35	0·09	-0·07	0·37	$2{\cdot}63x10^{-2}$	>0·05
4E-BP1	46	0·16	-0·52	0·73	$2{\cdot}32x10^{-1}$	>0·05	35	0·00	-0·69	1·45	$6{\cdot}33x10^{-1}$	>0·05
IL-8	45	0·12	-0·22	0·45	$2{\cdot}82x10^{-1}$	>0·05	35	0·06	-0·42	0·76	$4{\cdot}91x10^{-1}$	>0·05
CX3CL1	45	0·05	-0·10	0·24	$3{\cdot}02x10^{-1}$	>0·05	35	0·15	-0·16	0·48	$5{\cdot}57x10^{-2}$	>0·05
EN-RAGE	46	0·17	-0·52	0·72	$3{\cdot}02x10^{-1}$	>0·05	35	-0·09	-0·78	0·87	$8{\cdot}78x10^{-1}$	>0·05
CCL11	46	-0·03	-0·17	0·12	$3{\cdot}98x10^{-1}$	>0·05	35	0·00	-0·24	0·25	$9{\cdot}94x10^{-1}$	>0·05
FGF-5	46	-0·04	-0·14	0·08	$4{\cdot}04x10^{-1}$	>0·05	34	0·00	-0·11	0·09	$8{\cdot}79x10^{-1}$	>0·05
DNER	45	-0·03	-0·14	0·10	$4{\cdot}07x10^{-1}$	>0·05	35	-0·06	-0·30	0·12	$1{\cdot}00x10^{-1}$	>0·05
IL-7	46	0·09	-0·21	0·29	$4{\cdot}16x10^{-1}$	>0·05	35	-0·07	-0·16	0·33	$8{\cdot}91x10^{-1}$	>0·05
GDNF	45	-0·03	-0·21	0·19	$4{\cdot}88x10^{-1}$	>0·05	32	0·05	-0·18	0·20	$6{\cdot}38x10^{-1}$	>0·05
IL-10RA	44	-0·01	-0·18	0·11	$5{\cdot}13x10^{-1}$	>0·05	34	0·05	-0·09	0·20	$1{\cdot}79x10^{-1}$	>0·05
FGF-19	46	0·06	-0·47	0·60	$5{\cdot}30x10^{-1}$	>0·05	35	0·05	-0·84	0·62	$7{\cdot}28x10^{-1}$	>0·05
TWEAK	46	0·03	-0·17	0·23	$5{\cdot}30x10^{-1}$	>0·05	35	-0·01	-0·12	0·19	$8{\cdot}27x10^{-1}$	>0·05
LIF-R	46	0·03	-0·13	0·18	$6{\cdot}65x10^{-1}$	>0·05	35	0·02	-0·10	0·29	$1{\cdot}68x10^{-1}$	>0·05
OPG	45	0·03	-0·16	0·21	$7{\cdot}71x10^{-1}$	>0·05	35	-0·07	-0·26	0·16	$5{\cdot}01x10^{-1}$	>0·05
CCL25	46	0·02	-0·18	0·16	$9{\cdot}14x10^{-1}$	>0·05	35	0·00	-0·27	0·24	$9{\cdot}29x10^{-1}$	>0·05
NT-3	45	0·02	-0·18	0·18	$9{\cdot}29x10^{-1}$	>0·05	34	-0·04	-0·19	0·21	$6{\cdot}12x10^{-1}$	>0·05
MMP-10	46	0·03	-0·36	0·32	$9{\cdot}91x10^{-1}$	>0·05	35	0·11	-0·31	0·62	$3{\cdot}59x10^{-1}$	>0·05

Table S4. Association between ORR and inflammatory proteins changes from baseline at early (2-5 weeks after ICI initiation) and late (5-12 weeks after ICI initiation) follow-up visits. Protein change scores were estimated using Olink data in 46 (27 responders and 19 non-responders) and 35 (24 responders and 11 non-responders) patients with advanced melanoma at early and late follow-up time windows, respectively, and tested for association with ORR using a two-sided Wilcoxon test (p). Median change of $\log_2$-scaled protein levels, along with the lower (Q1) and upper (Q3) quartiles, are shown. A Wilcoxon empirical p-value (p_{emp}) was determined using multivariate permutation.

	**Early time window**											**Late time window**										
	**Responders**				**Non-Responders**							**Responders**				**Non-Responders**						
**Protein**	**N**	**median**	**Q1**	**Q3**	**N**	**median**	**Q1**	**Q3**	**mean $\log_2$-FC**	**p**	**p_{emp}**	**N**	**median**	**Q1**	**Q3**	**N**	**median**	**Q1**	**Q3**	**mean $\log_2$-FC**	**p**	**p_{emp}**
CX3CL1	26	0·13	0·02	0·35	19	-0·17	-0·40	0·12	-0·37	1·39x10^{-3}	>0·05	24	0·26	-0·08	0·56	11	-0·14	-0·35	0·13	-0·33	3·62x10^{-2}	>0·05
MMP-10	27	0·17	-0·08	0·36	19	-0·32	-0·48	-0·01	-0·50	7·03x10^{-3}	>0·05	24	0·18	-0·25	0·65	11	-0·20	-0·40	0·15	-0·39	2·24x10^{-1}	>0·05
IL-20RA	24	0·14	0·06	0·21	16	-0·06	-0·16	0·13	-0·14	4·15x10^{-2}	>0·05	20	-0·01	-0·12	0·09	11	-0·16	-0·25	0·20	-0·06	8·55x10^{-1}	>0·05
ST1A1	27	-0·14	-0·56	0·80	18	1·61	-0·52	1·79	0·67	5·85x10^{-2}	>0·05	24	-0·25	-1·09	0·48	11	-0·17	-0·27	0·55	0·36	3·70x10^{-1}	>0·05
MCP-4	27	0·25	0·02	0·45	19	-0·07	-0·22	0·40	-0·27	6·70x10^{-2}	>0·05	24	-0·01	-0·33	0·27	11	0·03	-0·15	0·20	0·06	9·03x10^{-1}	>0·05
CCL25	27	0·06	-0·09	0·19	19	0·00	-0·35	0·08	-0·22	8·18x10^{-2}	>0·05	24	0·08	-0·22	0·28	11	-0·11	-0·31	0·13	-0·09	3·90x10^{-1}	>0·05
SCF	26	0·02	-0·18	0·08	19	-0·22	-0·40	0·09	-0·17	1·11x10^{-1}	>0·05	23	-0·04	-0·24	0·22	11	-0·17	-0·42	0·35	0·17	9·71x10^{-1}	>0·05
IL-7	27	0·23	-0·15	0·32	19	-0·08	-0·18	0·09	-0·13	1·25x10^{-1}	>0·05	24	-0·07	-0·15	0·44	11	-0·07	-0·18	0·10	-0·14	6·62x10^{-1}	>0·05
DNER	26	0·01	-0·09	0·11	19	-0·09	-0·32	0·06	-0·13	1·27x10^{-1}	>0·05	24	-0·04	-0·30	0·14	11	-0·18	-0·27	0·04	-0·03	5·87x10^{-1}	>0·05
CD244	27	0·13	0·00	0·18	19	0·00	-0·22	0·27	-0·11	1·36x10^{-1}	>0·05	24	0·05	-0·16	0·26	11	0·08	-0·12	0·14	0·05	9·30x10^{-1}	>0·05
SIRT2	27	0·16	-0·61	0·76	19	1·09	-0·74	1·68	0·59	1·42x10^{-1}	>0·05	23	-0·14	-0·53	0·63	11	0·46	0·23	1·21	0·56	1·14x10^{-1}	>0·05
FGF-23	25	0·10	-0·12	0·24	16	0·17	0·07	0·35	0·13	1·47x10^{-1}	>0·05	20	-0·09	-0·34	0·30	11	-0·04	-0·11	0·25	0·07	6·40x10^{-1}	>0·05
TNFSF14	27	0·19	-0·10	0·62	19	0·68	0·12	1·20	0·32	1·55x10^{-1}	>0·05	24	0·10	-0·45	0·69	11	0·06	-0·61	0·54	0·00	8·75x10^{-1}	>0·05
CD6	27	0·29	0·01	0·63	19	0·10	-0·18	0·47	-0·27	1·68x10^{-1}	>0·05	24	0·49	0·08	0·62	11	0·22	0·02	0·60	-0·07	7·13x10^{-1}	>0·05
AXIN1	27	0·23	-0·33	0·56	19	1·00	-0·52	1·55	0·63	1·75x10^{-1}	>0·05	24	-0·12	-0·88	0·39	11	0·03	-0·45	0·43	0·57	4·30x10^{-1}	>0·05
EN-RAGE	27	-0·03	-0·50	0·40	19	0·47	-0·46	1·24	0·37	1·83x10^{-1}	>0·05	24	-0·08	-0·47	0·54	11	-0·13	-0·89	1·01	-0·09	7·40x10^{-1}	>0·05
IL-10	27	0·54	0·10	1·02	19	0·65	0·52	1·15	0·14	1·90x10^{-1}	>0·05	24	0·33	0·07	0·87	11	0·64	0·37	1·19	0·39	1·98x10^{-1}	>0·05
MCP-1	27	0·17	-0·04	0·35	19	0·01	-0·30	0·29	-0·20	1·90x10^{-1}	>0·05	24	-0·11	-0·40	0·14	11	-0·16	-0·35	0·41	0·06	7·93x10^{-1}	>0·05
CASP-8	27	0·00	-0·43	0·32	19	0·67	-0·40	1·01	0·35	1·98x10^{-1}	>0·05	24	0·10	-0·37	0·53	11	0·26	-0·34	0·80	0·07	7·40x10^{-1}	>0·05
FGF-19	27	0·09	-0·41	0·98	19	0·03	-0·63	0·37	-0·46	2·14x10^{-1}	>0·05	24	0·08	-0·59	0·61	11	-0·12	-0·86	0·53	0·19	9·30x10^{-1}	>0·05
OSM	27	0·40	0·15	0·64	19	0·74	0·21	1·12	0·14	2·14x10^{-1}	>0·05	24	0·23	-0·42	0·80	11	0·04	-0·81	0·45	-0·44	2·99x10^{-1}	>0·05
FGF-21	26	-0·38	-0·74	0·48	19	-0·39	-1·20	0·28	-0·39	2·21x10^{-1}	>0·05	24	-0·43	-0·91	0·09	11	-0·49	-0·78	0·42	-0·32	8·20x10^{-1}	>0·05
TNFB	27	0·27	0·14	0·35	19	0·18	-0·21	0·37	-0·13	2·22x10^{-1}	>0·05	24	0·27	-0·01	0·39	11	0·18	0·02	0·28	0·07	7·93x10^{-1}	>0·05

TRANCE	27	0·15	-0·10	0·50	19	-0·05	-0·37	0·44	-0·14	2·22x10^{-1}	>0·05	24	0·38	-0·20	0·51	11	0·31	-0·04	0·60	0·27	5·63x10^{-1}	>0·05
CST5	27	0·10	-0·04	0·25	19	-0·03	-0·19	0·23	-0·08	2·31x10^{-1}	>0·05	24	0·06	-0·08	0·26	11	0·10	-0·24	0·17	-0·10	5·63x10^{-1}	>0·05
Flt3L	27	0·32	0·05	0·49	19	0·14	-0·09	0·37	-0·16	2·49x10^{-1}	>0·05	24	0·14	-0·10	0·43	11	-0·13	-0·33	0·31	-0·04	4·72x10^{-1}	>0·05
IL-17C	25	0·21	-0·05	0·54	19	0·08	-0·10	0·27	-0·07	2·58x10^{-1}	>0·05	24	0·22	-0·30	0·68	11	0·22	-0·15	0·69	-0·12	7·93x10^{-1}	>0·05
NT-3	27	0·04	-0·11	0·18	18	-0·01	-0·23	0·17	-0·18	2·64x10^{-1}	>0·05	23	-0·04	-0·17	0·14	11	0·02	-0·31	0·22	-0·07	1·00E+00	>0·05
IL-15RA	26	0·22	0·10	0·34	19	0·18	-0·06	0·29	-0·08	2·99x10^{-1}	>0·05	24	0·28	0·11	0·39	11	0·13	0·02	0·28	-0·09	2·83x10^{-1}	>0·05
HGF	26	0·12	-0·04	0·33	19	0·23	-0·02	0·59	0·07	3·10x10^{-1}	>0·05	24	0·11	-0·20	0·51	11	0·01	-0·17	0·30	-0·26	2·68x10^{-1}	>0·05
FGF-5	27	0·00	-0·13	0·13	19	-0·06	-0·16	0·03	-0·05	3·30x10^{-1}	>0·05	23	-0·02	-0·10	0·07	11	0·03	-0·12	0·11	-0·04	1·00E+00	>0·05
LAP (TGFB1)	27	0·10	-0·14	0·39	19	-0·04	-0·22	0·22	-0·10	3·32x10^{-1}	>0·05	24	0·09	-0·13	0·31	11	0·05	-0·13	0·24	-0·04	8·75x10^{-1}	>0·05
CCL11	27	0·01	-0·16	0·12	19	-0·07	-0·34	0·16	-0·08	3·52x10^{-1}	>0·05	24	0·02	-0·21	0·22	11	-0·06	-0·37	0·28	-0·02	6·62x10^{-1}	>0·05
CXCL1	27	0·13	-0·03	0·28	19	0·30	0·02	0·48	0·05	3·52x10^{-1}	>0·05	24	0·15	-0·22	0·35	11	0·11	-0·11	0·33	-0·06	9·58x10^{-1}	>0·05
STAMBP	27	0·17	-0·25	0·63	19	0·56	-0·41	0·93	0·22	3·52x10^{-1}	>0·05	23	-0·01	-0·50	0·42	11	0·11	-0·04	0·72	0·44	2·14x10^{-1}	>0·05
TRAIL	27	0·04	-0·06	0·27	19	0·11	-0·02	0·36	0·07	3·52x10^{-1}	>0·05	24	0·13	-0·10	0·21	11	0·18	0·09	0·31	0·21	1·42x10^{-1}	>0·05
IL-6	26	-0·02	-0·30	0·34	19	0·31	-0·12	0·52	-0·24	3·68x10^{-1}	>0·05	24	-0·18	-0·98	0·18	11	0·01	-0·82	0·62	-0·20	8·47x10^{-1}	>0·05
CDCP1	26	0·20	-0·08	0·42	19	0·40	-0·14	0·63	0·11	3·93x10^{-1}	>0·05	23	0·14	-0·15	0·44	11	0·39	-0·03	0·58	0·28	2·28x10^{-1}	>0·05
4E-BP1	27	0·12	-0·64	0·56	19	0·19	-0·12	1·34	0·47	4·00x10^{-1}	>0·05	24	-0·10	-0·77	0·54	11	1·49	-0·50	1·98	1·07	1·07x10^{-1}	>0·05
CCL3	26	0·25	0·00	0·61	19	0·52	-0·06	1·18	0·14	4·05x10^{-1}	>0·05	24	0·21	-0·18	0·50	11	0·20	-0·72	0·83	-0·02	9·86x10^{-1}	>0·05
CD5	27	0·30	0·10	0·53	19	0·19	0·04	0·49	-0·14	4·52x10^{-1}	>0·05	24	0·39	0·07	0·60	11	0·25	0·14	0·51	-0·06	7·93x10^{-1}	>0·05
MCP-2	26	0·07	-0·02	0·28	19	0·01	-0·14	0·23	-0·06	4·59x10^{-1}	>0·05	23	-0·12	-0·27	0·12	11	-0·18	-0·40	0·11	-0·09	4·68x10^{-1}	>0·05
CXCL5	27	0·09	-0·07	0·27	19	0·20	-0·16	0·46	0·15	4·79x10^{-1}	>0·05	23	0·12	0·05	0·24	11	0·16	0·03	0·36	0·00	6·37x10^{-1}	>0·05
CCL19	27	0·39	0·21	0·96	19	0·69	0·21	1·23	-0·06	5·07x10^{-1}	>0·05	24	0·20	-0·25	0·88	11	0·35	-0·01	0·83	0·06	7·93x10^{-1}	>0·05
uPA	27	0·12	-0·03	0·31	19	0·19	-0·12	0·28	-0·07	5·51x10^{-1}	>0·05	24	0·05	-0·15	0·23	11	0·09	-0·11	0·27	0·07	5·87x10^{-1}	>0·05
TWEAK	27	0·04	-0·13	0·26	19	0·00	-0·19	0·19	-0·09	5·65x10^{-1}	>0·05	24	-0·03	-0·13	0·19	11	-0·01	-0·08	0·19	0·09	7·66x10^{-1}	>0·05
IL-8	26	0·06	-0·24	0·42	19	0·22	-0·09	0·43	0·06	5·77x10^{-1}	>0·05	24	0·04	-0·32	0·66	11	0·18	-0·51	0·94	0·04	8·75x10^{-1}	>0·05
CCL28	27	0·10	-0·12	0·26	19	0·00	-0·17	0·29	-0·16	6·27x10^{-1}	>0·05	24	0·10	-0·12	0·38	11	0·09	-0·01	0·22	0·02	6·62x10^{-1}	>0·05
LIF-R	27	0·03	-0·10	0·17	19	0·03	-0·16	0·14	-0·07	6·27x10^{-1}	>0·05	24	0·03	-0·08	0·32	11	-0·01	-0·18	0·18	-0·06	6·12x10^{-1}	>0·05
CCL20	27	0·13	-0·19	0·79	19	0·47	-0·53	0·98	-0·32	6·75x10^{-1}	>0·05	24	0·32	-0·43	1·02	11	0·39	-0·30	0·60	-0·33	8·20x10^{-1}	>0·05
CSF-1	27	0·07	0·00	0·14	19	0·15	-0·07	0·24	0·00	7·24x10^{-1}	>0·05	24	0·05	-0·07	0·20	11	0·07	-0·12	0·17	-0·07	6·62x10^{-1}	>0·05
IL-18	27	0·25	0·03	0·59	19	0·31	-0·12	0·59	-0·18	7·24x10^{-1}	>0·05	24	0·31	0·01	0·56	11	0·41	-0·16	0·60	-0·30	9·30x10^{-1}	>0·05
CD40	26	0·16	0·06	0·28	19	0·09	-0·22	0·46	0·00	7·24x10^{-1}	>0·05	24	0·11	-0·03	0·45	11	0·20	-0·03	0·37	0·02	9·30x10^{-1}	>0·05

IL-10RA	26	-0·01	-0·15	0·12	18	-0·04	-0·23	0·09	-0·02	7·32x10^{-1}	>0·05	23	0·06	-0·12	0·20	11	0·05	-0·02	0·18	-0·05	9·13x10^{-1}	>0·05
IL-18R1	26	0·11	-0·10	0·32	19	0·05	-0·21	0·29	-0·08	7·42x10^{-1}	>0·05	23	0·09	-0·19	0·25	11	0·09	-0·14	0·12	0·03	8·85x10^{-1}	>0·05
CXCL6	27	0·16	-0·05	0·32	19	0·20	-0·08	0·45	-0·02	7·57x10^{-1}	>0·05	24	0·08	-0·16	0·29	11	0·09	-0·05	0·24	0·03	6·12x10^{-1}	>0·05
CXCL11	27	0·67	0·21	1·10	19	0·74	0·25	1·71	-0·03	7·74x10^{-1}	>0·05	24	0·43	0·18	1·30	11	1·10	0·21	1·27	0·11	6·87x10^{-1}	>0·05
TNF	26	0·28	0·10	0·70	19	0·37	-0·18	1·02	-0·07	7·76x10^{-1}	>0·05	24	0·08	-0·22	0·58	11	0·54	0·15	0·77	0·16	3·52x10^{-1}	>0·05
IL-10RB	26	0·08	-0·01	0·19	18	0·15	-0·01	0·19	0·01	7·86x10^{-1}	>0·05	24	0·06	-0·02	0·21	10	0·14	-0·05	0·17	-0·02	8·67x10^{-1}	>0·05
IL-17A	26	0·36	0·08	0·54	18	0·40	0·10	0·67	-0·12	7·86x10^{-1}	>0·05	23	0·31	-0·10	0·60	10	0·26	-0·18	0·84	-0·43	9·54x10^{-1}	>0·05
CCL4	26	0·16	0·00	0·45	19	0·18	-0·15	0·77	0·00	7·94x10^{-1}	>0·05	24	-0·01	-0·15	0·33	11	-0·12	-0·26	0·21	-0·13	5·63x10^{-1}	>0·05
TNFRSF9	26	0·41	0·27	0·74	19	0·53	0·23	0·78	0·08	7·94x10^{-1}	>0·05	24	0·47	0·10	0·71	11	0·45	0·36	0·67	0·08	4·72x10^{-1}	>0·05
CD8A	27	0·21	-0·07	0·39	19	0·13	-0·18	0·62	0·12	8·08x10^{-1}	>0·05	24	0·17	-0·11	0·55	11	0·06	-0·08	0·62	0·04	9·58x10^{-1}	>0·05
CXCL10	27	0·72	0·20	1·42	19	0·83	0·22	1·53	-0·35	8·08x10^{-1}	>0·05	24	0·70	0·20	1·49	11	0·71	0·28	1·55	0·02	8·47x10^{-1}	>0·05
MMP-1	25	0·10	-0·12	0·22	18	-0·01	-0·11	0·19	0·05	8·33x10^{-1}	>0·05	23	-0·09	-0·22	0·05	11	0·05	-0·09	0·12	0·16	2·01x10^{-1}	>0·05
SLAMF1	27	0·24	-0·13	0·38	19	0·09	-0·04	0·41	0·00	8·42x10^{-1}	>0·05	24	0·09	-0·21	0·24	11	0·06	-0·25	0·41	-0·03	9·58x10^{-1}	>0·05
IL-12B	27	0·41	0·18	0·71	19	0·42	-0·08	0·79	-0·09	8·77x10^{-1}	>0·05	24	0·26	-0·15	0·67	11	0·52	0·22	0·97	0·33	1·74x10^{-1}	>0·05
TGF-α	27	0·24	-0·15	0·65	19	0·29	-0·02	0·63	-0·03	8·77x10^{-1}	>0·05	24	0·13	-0·44	0·66	11	-0·06	-0·52	0·27	-0·22	5·17x10^{-1}	>0·05
PD-L1	27	0·21	-0·03	0·48	19	0·27	-0·14	0·66	-0·06	9·12x10^{-1}	>0·05	24	0·16	-0·03	0·45	11	0·11	-0·05	0·29	-0·13	5·40x10^{-1}	>0·05
CXCL9	27	0·90	0·42	1·82	19	1·17	0·31	1·88	-0·29	9·30x10^{-1}	>0·05	24	0·88	0·40	1·78	11	1·49	0·91	1·96	0·05	4·30x10^{-1}	>0·05
VEGFA	27	0·11	-0·06	0·35	19	0·11	-0·17	0·50	0·01	9·47x10^{-1}	>0·05	24	0·05	-0·16	0·32	11	-0·09	-0·26	0·08	-0·12	2·38x10^{-1}	>0·05
GDNF	26	-0·05	-0·17	0·09	19	-0·01	-0·32	0·20	-0·07	9·55x10^{-1}	>0·05	22	0·16	-0·10	0·25	10	-0·19	-0·37	0·01	-0·23	4·28x10^{-2}	>0·05
CCL23	27	0·17	-0·11	0·50	19	0·13	-0·11	0·49	-0·01	9·65x10^{-1}	>0·05	24	0·12	-0·02	0·47	11	0·02	-0·12	0·60	-0·05	8·20x10^{-1}	>0·05
MCP-3	27	0·31	0·06	0·82	19	0·50	0·10	0·72	-0·18	9·65x10^{-1}	>0·05	24	0·12	-0·28	0·57	11	0·12	-0·16	0·74	0·03	8·47x10^{-1}	>0·05
IFN-γ	27	1·01	0·13	1·61	19	0·86	0·06	1·93	-0·15	9·82x10^{-1}	>0·05	24	0·49	-0·26	0·77	11	-0·19	-1·36	1·45	-0·38	7·66x10^{-1}	>0·05
ADA	26	0·10	-0·08	0·21	19	0·05	-0·17	0·40	0·01	9·91x10^{-1}	>0·05	23	0·17	-0·05	0·40	11	0·10	-0·13	0·44	0·07	9·71x10^{-1}	>0·05
OPG	26	0·03	-0·14	0·18	19	0·04	-0·28	0·22	-0·01	9·91x10^{-1}	>0·05	24	-0·06	-0·27	0·34	11	-0·08	-0·13	0·02	-0·16	6·36x10^{-1}	>0·05

Table S5. Association between ORR and serum levels of IL-6, HGF, and MCP-2 at early (2-5 weeks after ICI initiation) and late (5-12 weeks after ICI initiation) follow-up visits. Protein differences between 27 and 24 responders, and 19 and 11 non-responders were assessed using Wilcoxon's test (p) on Olink data at early and late follow-up time windows, respectively. Median, lower (Q1) and upper (Q3) quartiles of log_2-scaled protein levels among responders and non-responders are shown. A Wilcoxon empirical p-value (p_{emp}) was determined using multivariate permutation.

		**Responders**				**Non-Responders**						
**Protein**	**window**	**N**	**median**	**Q1**	**Q3**	**N**	**median**	**Q1**	**Q3**	**mean log_2-FC**	**p**	**p_{emp}**
HGF	early	26	9·71	9·40	9·92	19	10·18	9·90	10·48	0·34	$2·75x10^{-3}$	$9·40x10^{-3}$
IL-6	early	27	2·84	2·40	3·71	19	3·61	3·08	4·43	0·31	$2·32x10^{-2}$	$4·66x10^{-2}$
MCP-2	early	27	9·76	9·46	10·23	19	10·08	9·74	10·64	0·43	$3·31x10^{-2}$	$3·38x10^{-2}$
IL-6	late	24	2·82	2·33	3·27	11	3·65	3·26	3·87	0·68	$1·33x10^{-2}$	$3·94x10^{-2}$
MCP-2	late	24	9·58	9·20	10·18	11	9·97	9·67	10·62	0·50	$1·52x10^{-1}$	>0·05
HGF	late	24	9·72	9·50	10·30	11	9·95	9·63	10·10	0·02	$4·72x10^{-1}$	>0·05

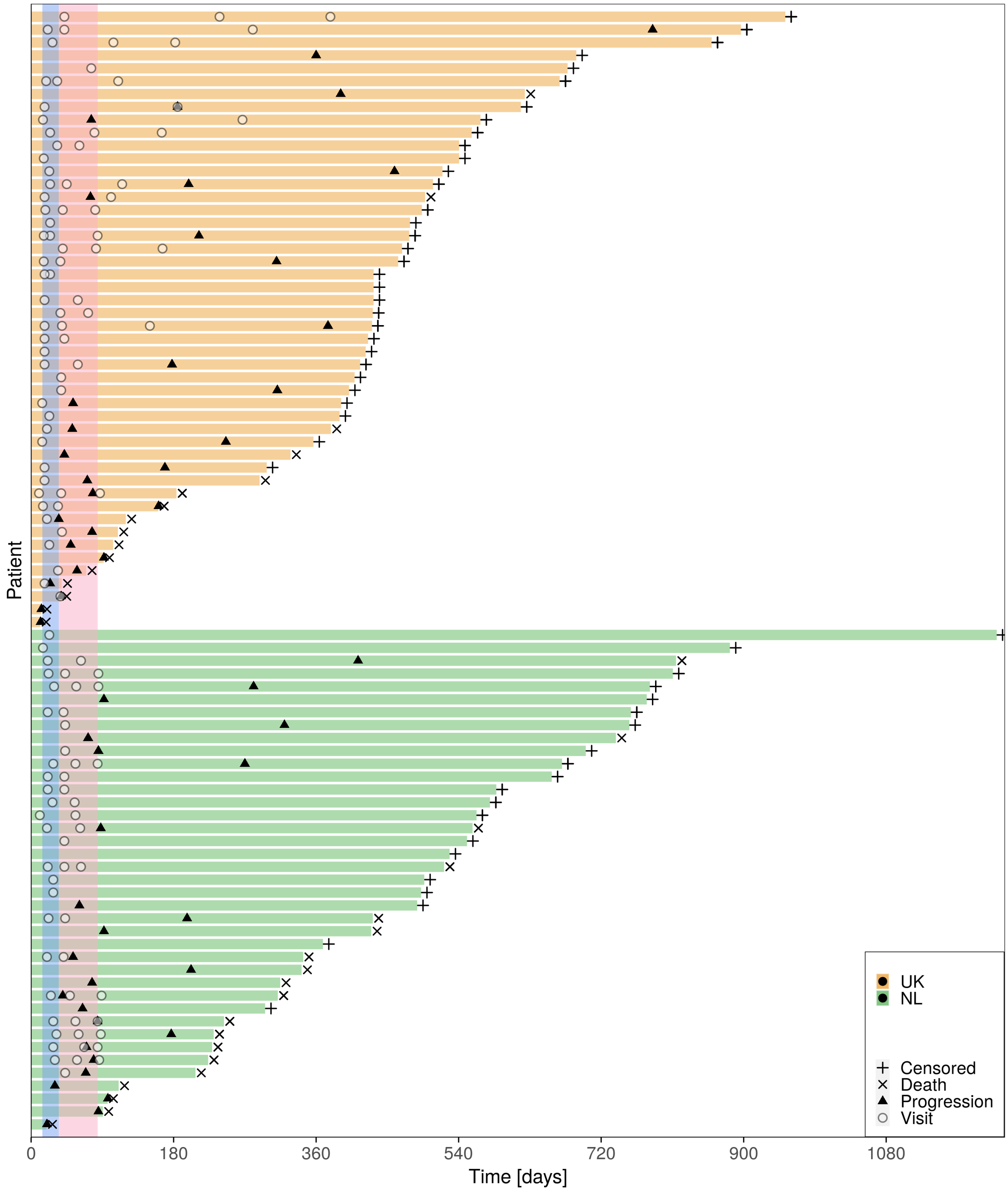

Figure S1. Swimmer plot for 87 patients with advanced melanoma. Day 0 indicates the day of ICI treatment initiation. Baseline samples were collected on average seven days before starting ICI. Only visits carried out during the early (blue, 2-5 weeks after ICI initiation) and late (pink, 5-12 weeks after ICI initiation) follow-up time windows were included in the study.

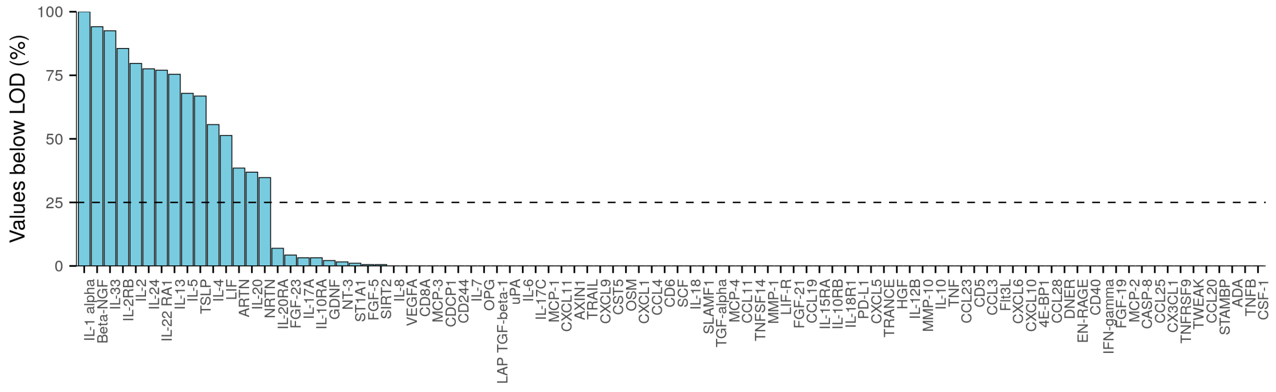

Figure S2. Bar plot showing the fraction of values below the limit of detection (LOD) among 92 inflammatory proteins measured in 187 serum samples using the Olink Inflammation panel. Proteins with >25% values below LOD (horizontal dashed line) were discarded.

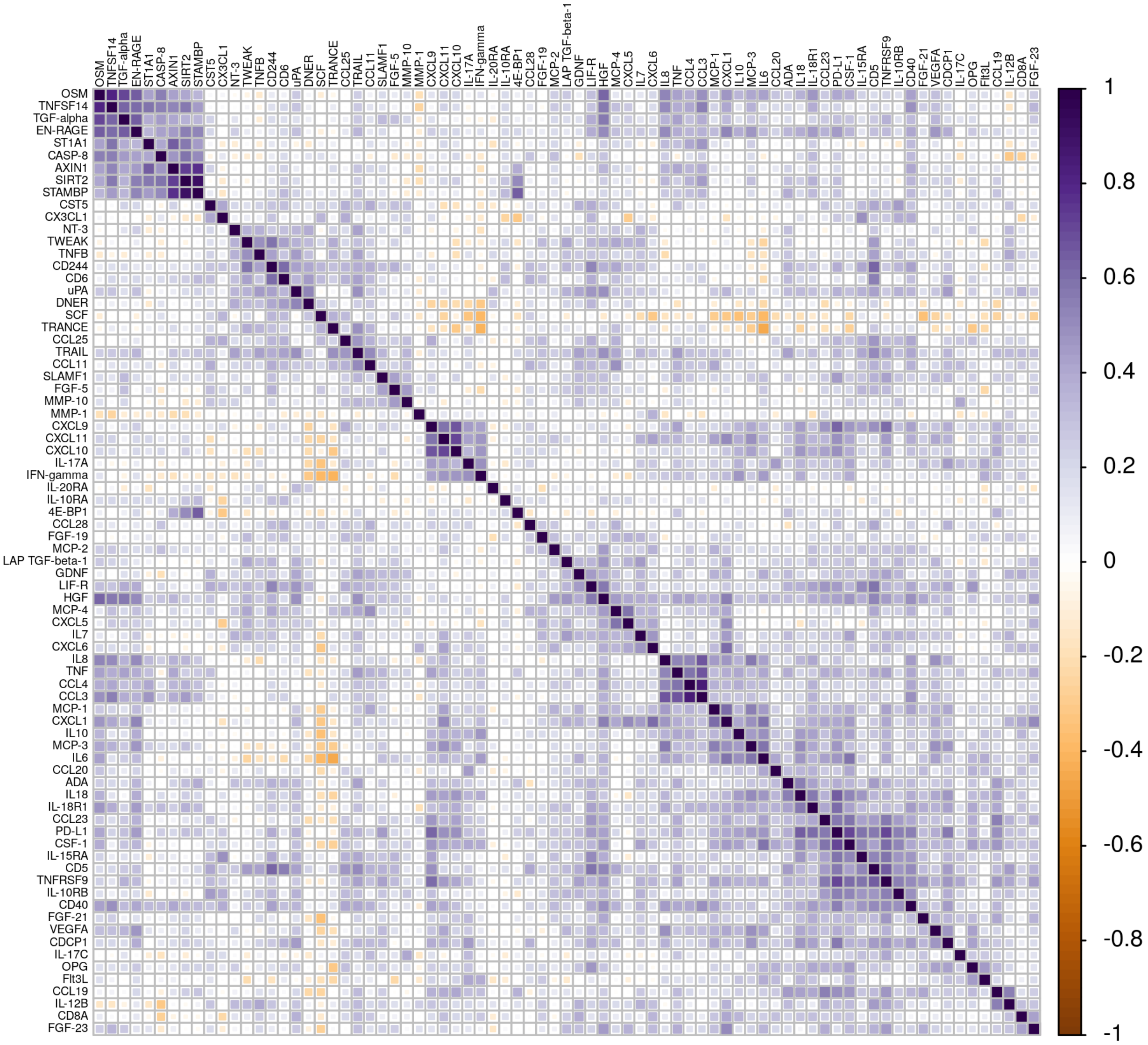

Figure S3. Heatmap showing baseline correlation of 77 inflammatory proteins measured using the Olink inflammation panel in serum samples from 87 patients with advanced melanoma.

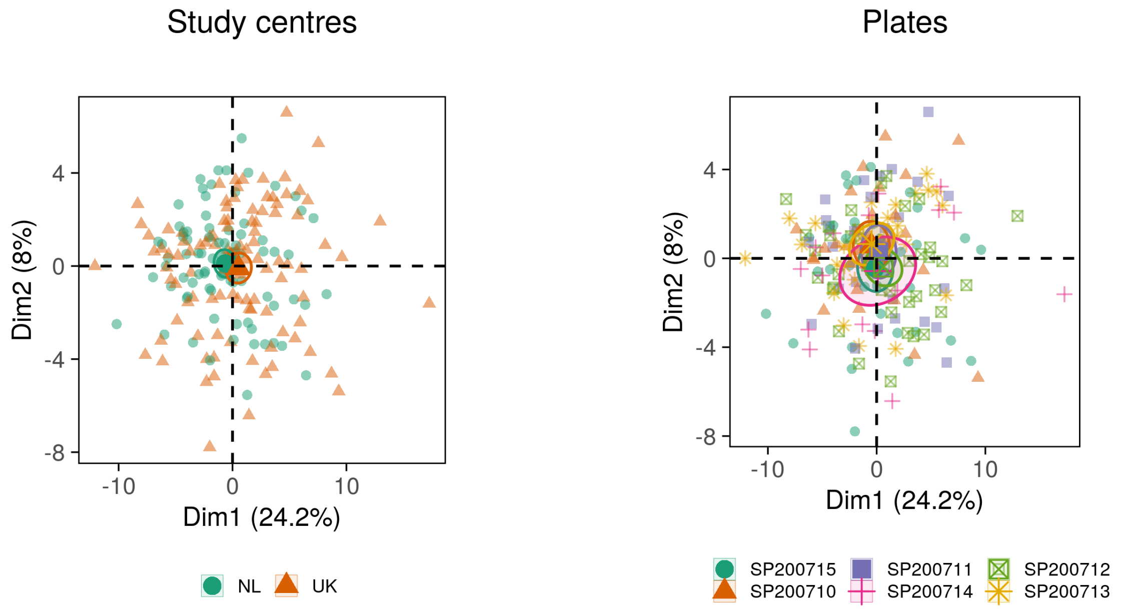

Figure S4. Scatterplot of the first two principal components assessed on Olink proteomic data measured in 187 serum samples from 87 patients with advanced melanoma. Individual data points are colour-coded according to the study centre (left panel) or assay plate (right panel).

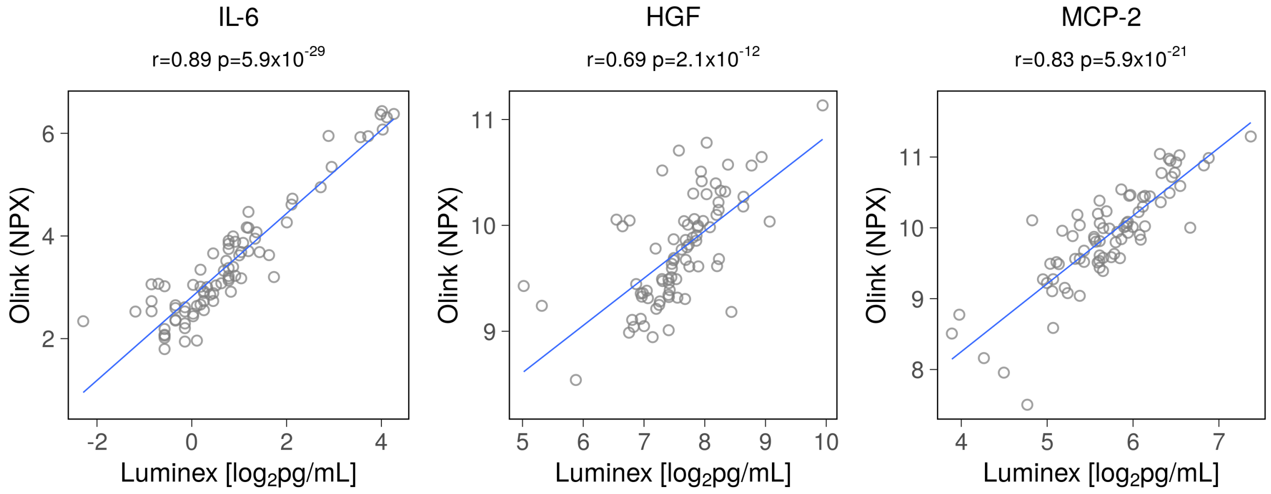

Figure S5. Correlation plot of Olink and Luminex data in 81 patients with advanced melanoma. Spearman correlation coefficients and p-values are shown. Olink data is in an arbitrary log_2-scaled NPX (Normalised Protein eXpression) unit.

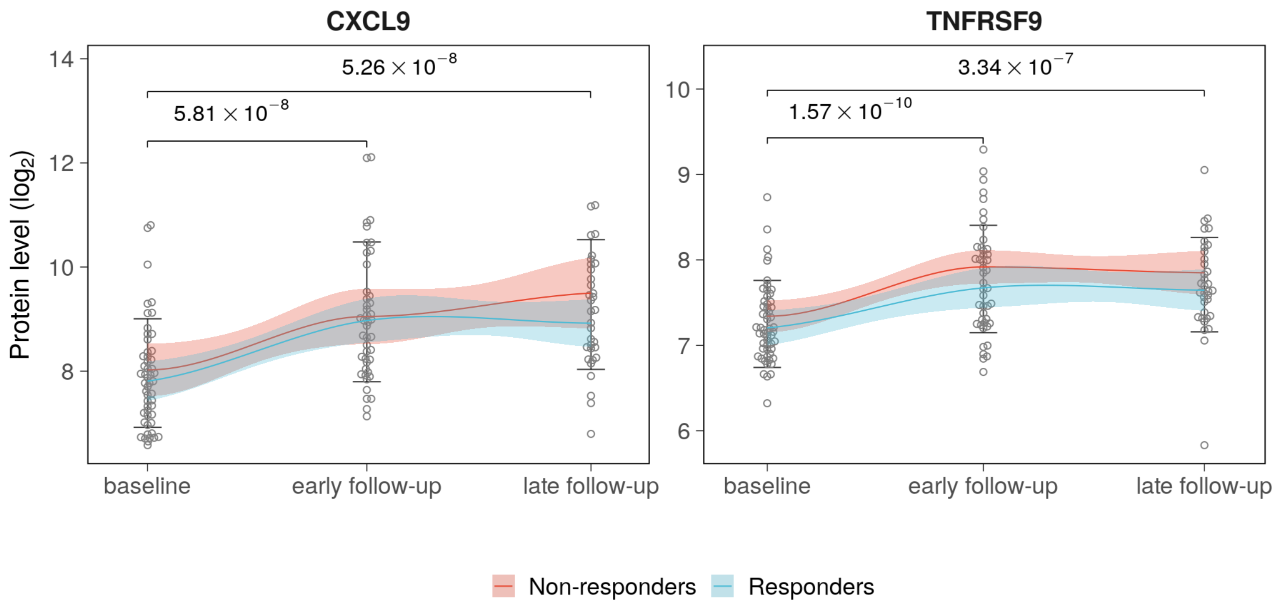

Figure S6. Distribution of CXCL9 and TNFRSF9 serum levels in patients with advanced melanoma at baseline and after ICI initiation at early and late follow-up time windows. The early follow-up window comprises follow-up samples collected within 2-5 weeks after starting treatment (average 4 weeks), while the late follow-up window comprises follow-up samples collected within 5-12 weeks after starting treatment (average 8 weeks). P-values were calculated using a two-sided Wilcoxon test for paired data. For responders and non-responders, 95% CIs of the smoothed conditional mean are shown.

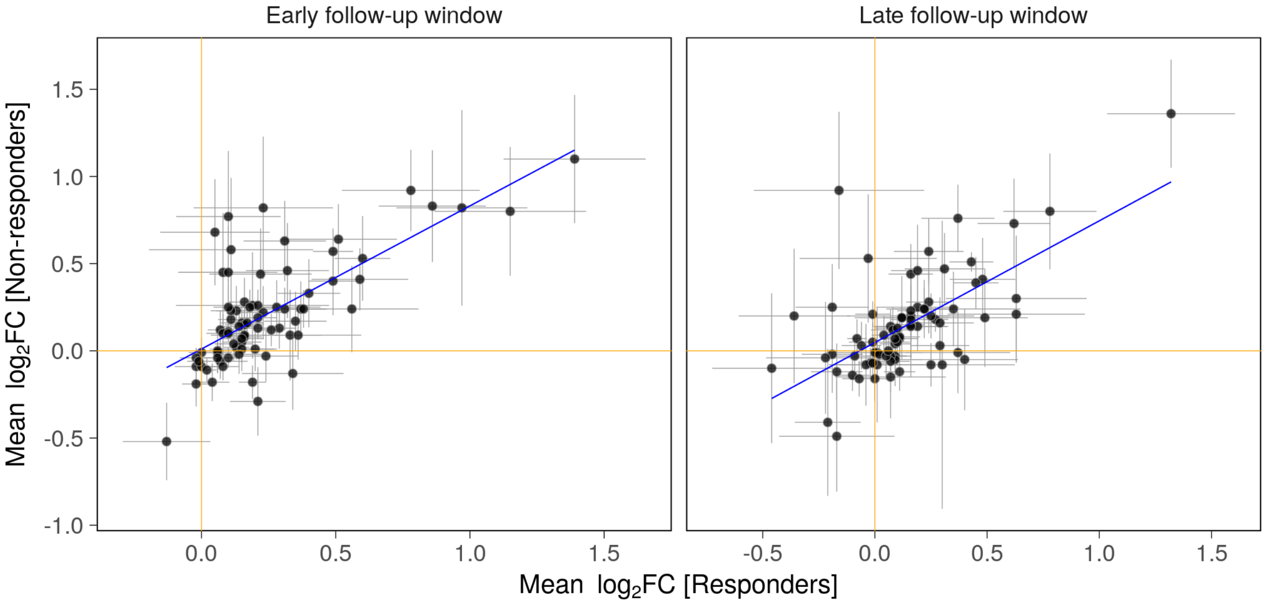

Figure S7. Scatterplot showing correletation of change scores of 77 inflammatory proteins from baseline at early and late follow-up visits between responders and non-responders. The early and late follow-up windows comprise follow-up samples collected within 2-5 weeks and 5-12 weeks after ICI initiation, respectively. Each point indicates the mean longitudinal change from baseline to follow-up of $\log_2$-scaled protein levels, while lines represent the standard deviation.

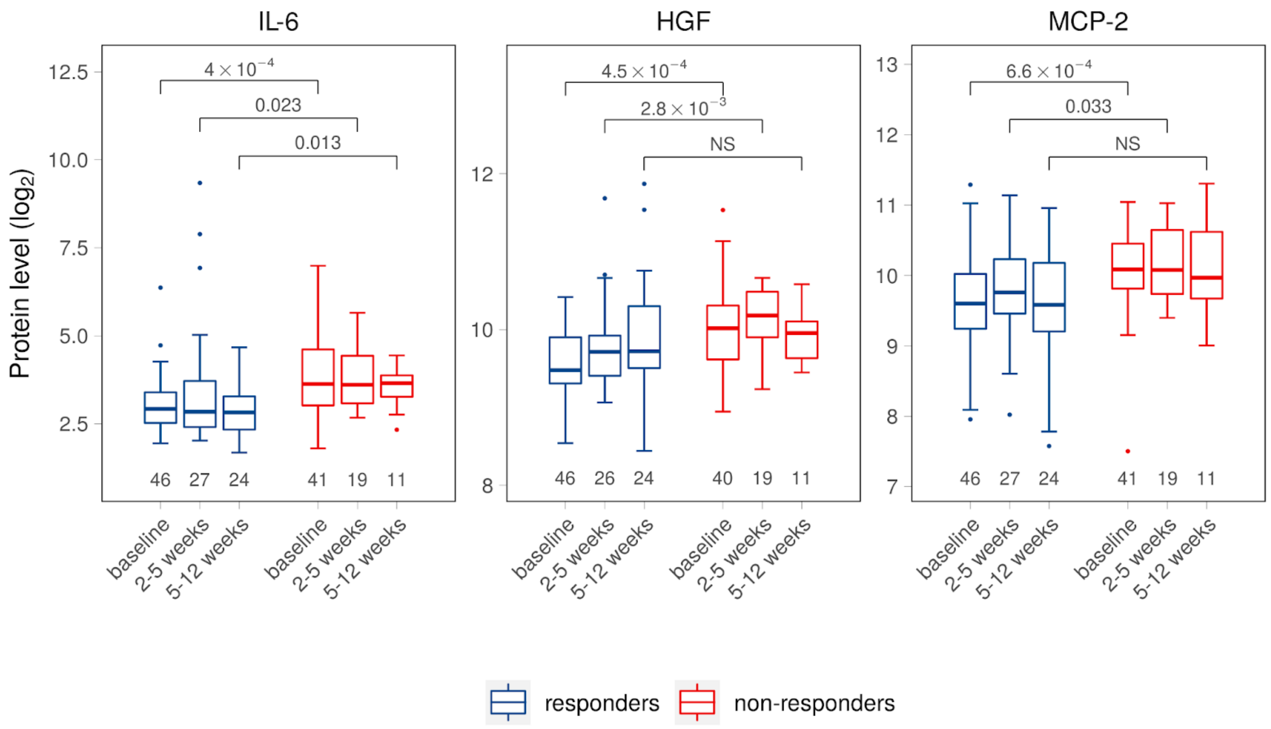

Figure S8. Distribution of IL-6, HGF, and MCP-2 serum levels in patients with advanced melanoma at baseline and after ICI initiation at early and late follow-up time windows. The early follow-up window comprises follow-up samples collected within 2-5 weeks after starting treatment, while the late follow-up window comprises follow-up samples collected within 5-12 weeks after starting treatment. P-values were calculated using the Wilcoxon test. Number of patients with serum samples available at each time window is shown. *NS*: p>0·05.

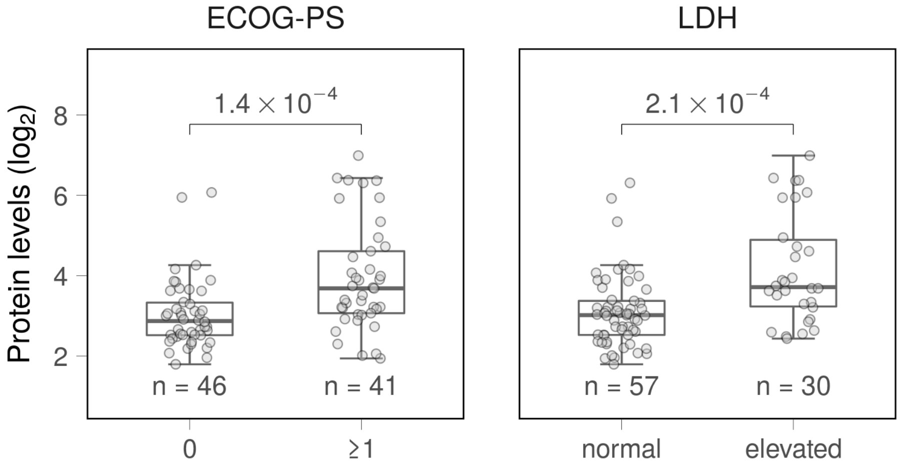

Figure S9. Distribution of pre-treatment serum levels of IL-6 (Olink data) according to ECOG-PS (left panel) and LDH levels (right panel).

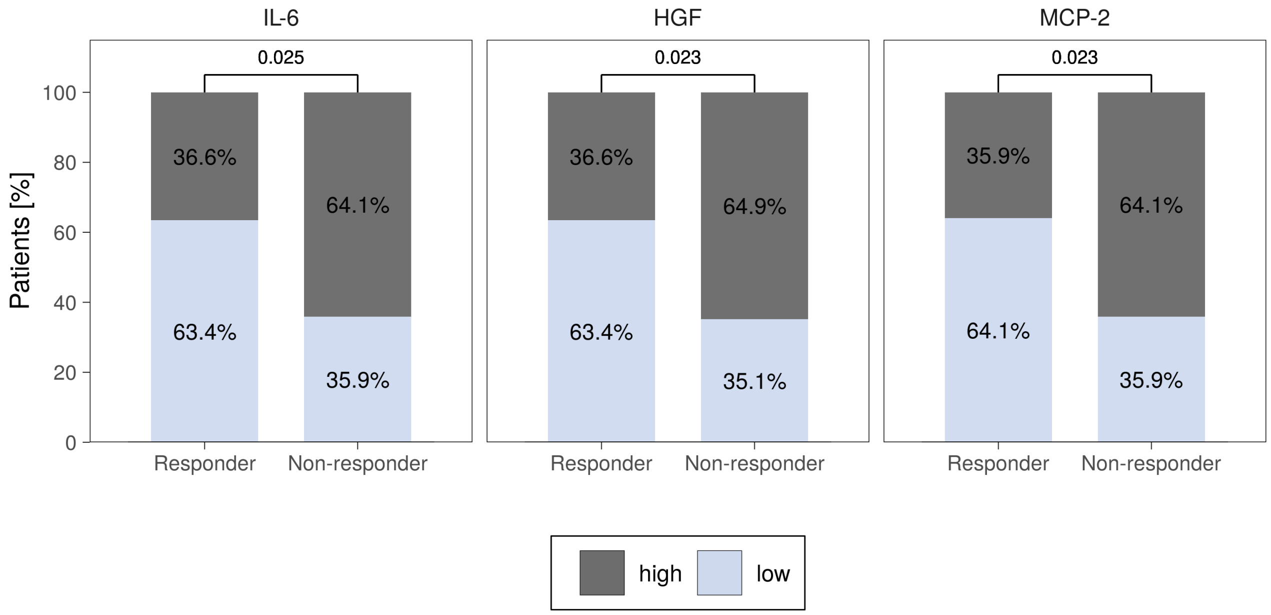

Figure S10. Bar plot showing the percentages of patients with advanced melanoma presenting with high (above the median, grey) or low (below the median, light blue) pre-treatment levels of IL-6, HGF, and MCP-2 stratified according to response to ICI. Fisher's p-values are shown.

References

1. Blackford JU, Salomon RM, Waller NG. Detecting change in biological rhythms: a multivariate permutation test approach to Fourier-transformed data. Chronobiol Int. 2009 Feb;26(2):258–81.
